# A Phase I study of Milademetan (DS3032b) in combination with low dose cytarabine with or without venetoclax in acute myeloid leukemia: Clinical safety, efficacy, and correlative analysis

**DOI:** 10.1038/s41408-023-00871-1

**Published:** 2023-06-29

**Authors:** Jayastu Senapati, Muharrem Muftuoglu, Jo Ishizawa, Hussein A. Abbas, Sanam Loghavi, Gautam Borthakur, Musa Yilmaz, Ghayas C. Issa, Samuel I. Dara, Mahesh Basyal, Li Li, Kiran Naqvi, Rasoul Pourebrahim, Elias J. Jabbour, Steven M. Kornblau, Nicholas J. Short, Naveen Pemmaraju, Guillermo Garcia-Manero, Farhad Ravandi, Joseph Khoury, Naval Daver, Hagop M. Kantarjian, Michael Andreeff, Courtney D. DiNardo

**Affiliations:** 1 Department of Leukemia, MD Anderson Cancer Center, Houston, TX USA; 2 Department of Hematopathology, MD Anderson Cancer Center, Houston, TX USA

**Keywords:** Translational research, Acute myeloid leukaemia

## Abstract

In *TP53* wild-type acute myeloid leukemia (AML), inhibition of MDM2 can enhance p53 protein expression and potentiate leukemic cell apoptosis. MDM2 inhibitor (MDM2i) monotherapy in AML has shown modest responses in clinical trials but combining options of MDM2i with other potent AML-directed agents like cytarabine and venetoclax could improve its efficacy. We conducted a phase I clinical trial (NCT03634228) to study the safety and efficacy of milademetan (an MDM2i) with low-dose cytarabine (LDAC)±venetoclax in adult patients with relapsed refractory (R/R) or newly diagnosed (ND; unfit) *TP53* wild-type AML and performed comprehensive CyTOF analyses to interrogate multiple signaling pathways, the p53-MDM2 axis and the interplay between pro/anti-apoptotic molecules to identify factors that determine response and resistance to therapy. Sixteen patients (14 R/R, 2 N/D treated secondary AML) at a median age of 70 years (range, 23–80 years) were treated in this trial. Two patients (13%) achieved an overall response (complete remission with incomplete hematological recovery). Median cycles on trial were 1 (range 1–7) and at a median follow-up of 11 months, no patients remained on active therapy. Gastrointestinal toxicity was significant and dose-limiting (50% of patients ≥ grade 3). Single-cell proteomic analysis of the leukemia compartment revealed therapy-induced proteomic alterations and potential mechanisms of adaptive response to the MDM2i combination. The response was associated with immune cell abundance and induced the proteomic profiles of leukemia cells to disrupt survival pathways and significantly reduced MCL1 and YTHDF2 to potentiate leukemic cell death. The combination of milademetan, LDAC±venetoclax led to only modest responses with recognizable gastrointestinal toxicity. Treatment-induced reduction of MCL1 and YTHDF2 in an immune-rich milieu correlate with treatment response.

## Introduction

Outcomes in patients with relapsed/refractory (R/R) acute myeloid leukemia (AML) remain poor. The wild-type (WT) *TP53 (TP53*^*wt*^*)* gene translation of normal p53 protein expression contributes significantly to therapeutic efficacy in AML, by enabling apoptosis of leukemic cells exposed to chemotherapy [1]. Multiple mechanisms are involved in maintaining the intricate balance of *TP53* gene expression and functional p53 protein levels [2, 3]. In steady-state normal cells, murine double minute protein 2 (MDM2) interacts with p53^wt^ protein and causes ubiquitin-mediated degradation of the latter, thereby limiting excessive p53 protein levels [4]. Translating this biological potential of MDM2 inhibition into therapy increases p53 protein expression and mediates antileukemic effects in *TP53*^*wt*^ AML and other cancer cells [5]. Early clinical investigation with MDM2 inhibitors (MDM2i) as monotherapy has demonstrated only modest benefits [6]. Based on preclinical data [7], venetoclax was combined with idasanutlin (an MDM2i) in a phase Ib clinical trial that included patients ≥ 60 years of age R/R AML or newly diagnosed (ND) treated secondary AML [8, 9]. The composite complete remission rate at the recommended phase 2 dose (RP2D) was 34%. The regimen was reasonably well tolerated; though 87% of study patients experienced diarrhea (24). Milademetan (DS3032b) is an orally active MDM2i, that has been shown to disrupt the MDM2-p53 axis and stabilize p53 levels in cells [10]. Preliminary results from a phase I dose-finding study of milademetan monotherapy that included 38 patients with R/R AML or high risk myelodysplastic syndrome (MDS) showed modest responses (8%), though >50% of patients had a bone marrow (BM) blast reduction at the end of the first cycle [11]. The majority of grade ≥ 3 treatment-emergent adverse events were gastrointestinal or hematologic. To evaluate whether milademetan may demonstrate clinical synergy with cytarabine with/without venetoclax, we conducted a clinical trial to evaluate the safety and determine the optimal combination dose of milademetan with low dose cytarabine (LDAC) ± venetoclax in patients with AML and *TP53*^*wt*^. Extensive correlative analyses to determine factors that affected response to our MDM2i based combination therapy were performed.

## Methods

### Patients and treatment

This investigator-initiated trial was approved by the MD Anderson Institutional Review Board, registered on ClinicalTrails.gov (NCT03634228) and conducted in accordance with the Declaration of Helsinki. Eligible patients were adults ≥ 18 years of age with a diagnosis of AML (R/R AML, or ND AML not eligible for intensive chemotherapy due to age or comorbidities) according to World Health Organization 2016 criteria. Key exclusion factors included the presence of a *TP53* mutation and chromosome 17p aberration, prior treatment with an MDM2i and the presence of central nervous system leukemia (Supplemental study protocol). The Phase I portion used a 3 + 3 Bayesian study design to identify the RP2D combination dose. Four dose levels of milademetan with LDAC, ± venetoclax were tested (Supplementary Fig. 1). Based on safety and tolerability data from a phase I dose escalation study of milademetan in patients with hematological malignancies, the starting dose of milademetan was 120 mg (dose level 0), in combination with LDAC [11]. Eighteen patients were planned to be enrolled in the phase I portion of the study. Toxicity was graded according to the NCI CTCAE, v5.

### Evaluations and correlative analysis

BM and peripheral blood (PB) samples for disease assessment were obtained at baseline and at the End of Cycle (EOC)1. European LeukemiaNet (ELN) 2017 criteria were used for response assessment [12]. All patients had baseline next-generation sequencing (NGS) using an 81 gene myeloid panel as the standard of care, including *TP53* genotyping, at the MDACC CLIA certified lab. P53 immunohistochemistry (IHC) was evaluated on available baseline and follow-up BM specimens, as previously published [13]. Serial PB and BM samples were collected from patients for cytometry by time-of-flight (CyTOF) analysis using a 51-parameter, leukemia-focused CyTOF panel. A comprehensive analysis of sequentially collected samples was performed with the aim to interrogate a multitude of signaling pathways, including the p53-MDM2 axis, the abundance of pro/anti-apoptotic molecules, and adaptive mechanisms and alterations in the leukemia proteomic landscape (Supplemental methods).

### Statistical analysis

The primary objective of this study was to evaluate the safety and determine the RP2D of milademetan (Phase I) and efficacy (by ELN 2017 criteria – Phase 2) of the combination therapy in both frontline and R/R AML patient population. Secondary and exploratory objectives are detailed in the supplemental study protocol.

## Results

A total of 21 patients were screened for the study, of whom 16 patients met all inclusion and exclusion criteria and were treated in phase I. The baseline characteristics of the patients are shown in Table 1. The median age of the patients was 70 years (range, 23–80). Two patients (12.5%) had ND, treated secondary AML, having received 2 and 3 lines of therapy for a prior diagnosis of MDS. The remaining 14 patients (87.5%) had R/R AML and had received 3 median prior lines of therapies (range, 1–7). Six patients (38%) had also undergone prior allogeneic stem cell transplantation (SCT) for AML (Supplementary Fig. 2). Thirteen (93%) patients with R/R AML had previous exposure to venetoclax. Per study protocol, all patients were *TP53*^*wt*^ at the time of enrollment.

**Table 1:** Baseline characteristics of the study patients.

Baseline characteristics		*N*, median (%) [range] (*N*=16)
Age (years)		70 [20–30]
Gender	Female	11 (69)
Diagnosis	R/R AML	14 (88)
	ND secondary AML	2 (12)
BM blasts (%)		36 [8–94]
Cytogenetics	Core Binding Factor [t (8;21)]	1 (6)
	Diploid karyotype	4 (25)
	Adverse karyotype	6 (38)
	‐ Complex	4 (25)
	Miscellaneous	5 (31)
ELN 2017 risk category	Favorable risk	1 (6)
	Intermediate risk	5 (31)
	Adverse risk	10 (63)
Mutations	*ASXL1*	4 (25)
	*DNMT3A*	3 (18)
	*TET2*	4 (25)
	*RUNX1*	5 (31)
	*NRAS/KRAS*	7 (44)
	*PTPN11*	3 (18)
	*WT1*	2(12)
Prior lines of therapy		3 [1–6]
	Prior venetoclax	13 (93)
	Prior allo-SCT	6 (38)

*N* number, *R/R* relapsed refractory, *ND* newly diagnosed, *ELN* European LeukemiaNet, *allo-SCT* allogeneic stem cell transplantation.

### Safety and efficacy

Three patients were treated at dose level 0 (19%), while 6 (37%) and 7 (44%) patients were treated at “triplet” dose levels 1 and 2, respectively. Dose level 2 was considered the maximum tolerated dose (MTD). All patients experienced at least one treatment-emergent adverse event (AE); one patient experienced an attributable grade ≥ 3 AE of diarrhea at dose level 2 which was considered a dose-limiting toxicity. Most of the attributable AE were gastrointestinal of grades 1-2. Additional gastrointestinal AE included grade 3 infectious enterocolitis in 4 patients (25%) and grade 3 proctitis and ileus in one patient each. Of the non-attributable AE, the most common were infections: 10 patients (63%) had lung infections (all grade 3), 6 patients (38%) had sepsis (5 grade 3 and 1 grade 4) and one patient each experienced grade 3 salivary gland infection, skin infection and hepatic infection (Supplemental Table 1).

Patients received a median of 1 cycle (range, 1–4) in the study. Five patients (31%) received >1 cycle of therapy and two patients (12.5%), both R/R AML, achieved an overall response [both attained CR with incomplete hematologic recovery (CRh); 1 each at dose level 1 and 2] after failure of a prior venetoclax containing regimen. Both patients attained MRD negative status by flow cytometry (at 1 in 10^4^ sensitivity), occurring end of cycle (EOC) 2 and 3. The first patient discontinued therapy in an ongoing leukemia-free state after cycle 3 because of protracted cytopenia. After recovery of counts at cycle 3, day 144, the patient was started on decitabine maintenance and continues to remain in MRD-negative CR at 18 months of follow-up. The other responding patient also received 3 cycles of therapy and discontinued study treatment due to protracted cytopenias; he died in remission after 8 months of follow-up (C3 D139) from neutropenic infections. In addition, one non-responding patient was taken off study after C1 due to recurrent infections precluding initiation of C2. The EOC1 BM assessment showed persistent disease with 50% blasts, but while on supportive care after discontinuing study treatment, and without any intervening therapy, he attained CRh at around D100 post C1. Two additional patients experienced a > 50% BM blast reduction at the end of C1. At a median follow-up of 11 months, no patients remained on study treatment and 11 patients had died. The median OS of the whole cohort was 2.4 months. Seven patients died on treatment; none of the deaths were attributable to a direct toxicity from the study drug combination. The cumulative 30 days and 60 days mortality were 6% and 31% respectively. The most common cause for study protocol discontinuation was the absence of response (*n* = 7) (Supplementary Fig. 3). In view of the modest Phase Ib response rates, the phase 2 expansion portion of the study was not conducted.

### Correlative analysis

P53 IHC was performed on baseline BM samples from 11 patients, all of whom had WT expression pattern. Assessment of paired BM samples collected post C1 demonstrated acquisition of mutant p53 expression (confirmed at low level by NGS: *TP53* VAF < 5%) in one of eight patients evaluated. This patient was a non-responder and went on to receive a total of 3 cycles of therapy at dose level +1.

**Fig. 1:**
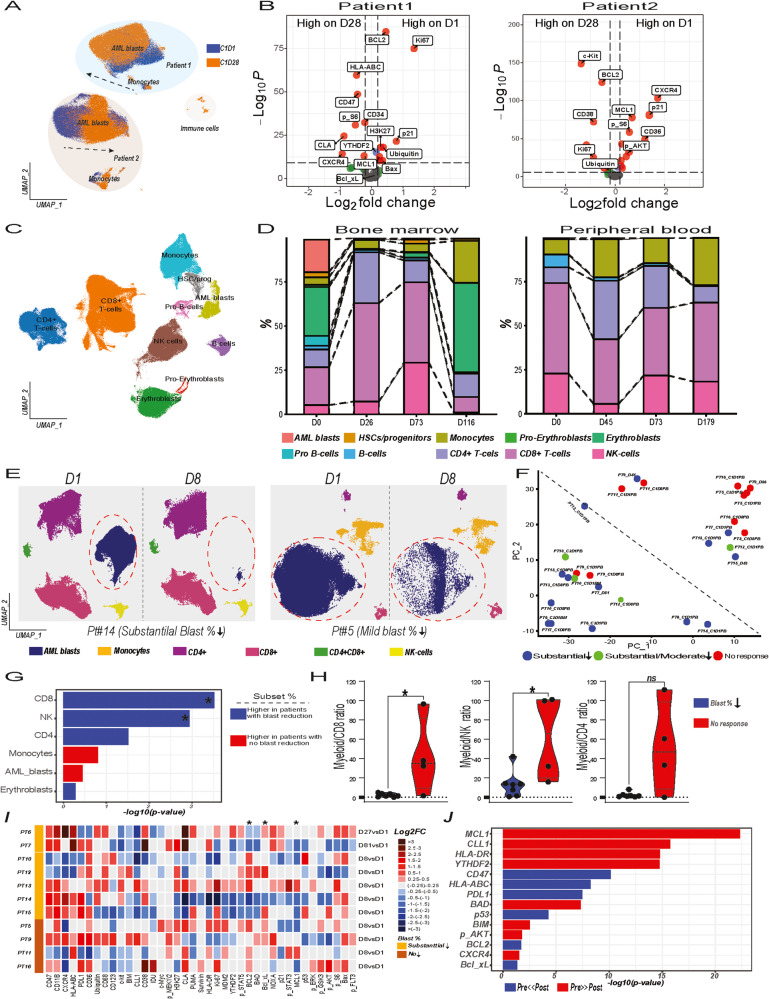
Comprehensive Single-Cell Proteomic Analysis of AML Patients Treated with Milademetan, LDAC±Venetoclax Therapy Reveals Unique Proteomic Profiles and Therapy-Induced Alterations. **A** 20,000 cells from pre- and post-treatment PB samples collected from patients, Pt1 and Pt2, were subjected to UMAP dimension reduction and projected in two dimensions. Pre-(orange) and post-treatment (blue) samples are color-coded. Arrows show the direction of proteomic shift induced by doublet therapy in myeloid cells (AML blasts and monocytes). **B** Volcano plot showing the differentially expressed proteins in PB leukemia cells from Pt1 (left) and Pt2 (right) assessed on D1 vs D28. Features shown on the right-hand side are detected at higher levels on D1. The threshold in the volcano plot was log10 adjusted p > 12 and log 2-fold change >0.25. **C** Cells from serially collected PB and BM samples from Pt8 with CR were pooled and subjected to UMAP dimension reduction. UMAP plots show cell subsets detected in leukemia compartments. Colors indicate cell type. **D** Stacked bar plots summarize the subset frequencies in serial PB (left) and BM (right) samples. **E** UMAP plots show cell subsets detected in leukemia compartments on D1 and D8 in a patient with substantial blast reduction (Pt14) (left panel) and a non-responding (right panel) AML patient (Pt5). Colors indicate cell type. **F** Cell type frequencies shown in panel B were used for UMAP dimension reduction to map similarities and dissimilarities with regards to cell type composition. The color indicates the response type achieved with triplet therapy. **G** Bar chart shows differential abundance analysis. Baseline cell type frequencies detected through UMAP analysis in patients with substantial blast reduction vs. those with no or minor blast reduction were compared (*p < 0.05). Blue and red colors indicate higher median cell frequencies in patients with substantial blast reduction (blue) and no or minor reduction (red), respectively **H**) Violin plots show myeloid to CD8 + T-cell (left), NK-cell (middle) and CD4 + T-cell (right) ratios in responders vs non-responders. **I** Heatmap shows log2 fold changes (FC) for the assessed markers using CyTOF data (columns) for eleven patients that received triplet therapy and failed to achieve a clinical response. Patients are stratified per changes in blast counts in **A** and supplemental Fig. 5A and proteomic features of D8 samples or earliest available samples were compared to those of baseline samples to calculate FCs. Increase and decrease in protein expression in AML blasts identified through UMAP analysis shown in shades of red and blue, respectively. FCs <0.25 are shown in gray. * denotes several selected markers differentially regulated after triplet therapy. **J** Bar chart shows top differentially expressed features after triplet therapy by comparing protein expression levels of pre- and post-treatment samples (*n* = 11) shown in **H**. Blue and red colors indicate high and low expression levels of the indicated markers in post-therapy samples, respectively.

CyTOF analysis of PB and BM samples from AML patients treated with Milademetan, LDAC ±Venetoclax therapy was performed to interrogate the alterations in proteomic landscape and potential resistance mechanisms (Supplementary Fig. 4A). Unsupervised clustering of pre- and post-therapy samples from patients receiving doublet therapy (DT) demonstrated distinct proteomic profiles and heterogeneity (Fig. 1A and Supplemental Fig. 4B–D). DT induced alterations in the proteomic landscape but did not significantly reduce leukemia fractions (Fig. 1B and Supplemental Fig. 4E–M). Differential expression analysis revealed distinct therapy-driven alterations in the proteomic landscape across patients, including downregulation of BCL2 and MCL1 in Pt1 and suppression of multiple signaling pathways and profound MCL1 reduction in Pt2 (Fig. 1B and Supplemental Fig. 4H–K). Therapy-induced downregulation of MCL1 and suppression of p21 expressing leukemia cells emerged as shared features among patients (Supplemental Fig. 3L–M).

Two (Pt4 and Pt8) out of thirteen patients treated with triplet therapy (TT) achieved CRh, and single-cell proteomic analysis was performed to characterize the leukemia landscape in responders. CyTOF analysis revealed that AML blasts were situated in close proximity to healthy progenitor cells in Pt8, and the preserved healthy BM microenvironment may have prevented leukemia cell expansion in this patient (Fig. 1C). Triplet therapy induced substantial reduction in leukemia cells and proteomic shifts in the AML proteomic landscape (Fig. 1D and Supplemental Fig. 5A). The surviving AML blasts uniquely rewired their profiles, characterized by increased activity of AKT and mTOR pathways, upregulation of YTHDF2, a transcription factor regulating RNA metabolism and counteracting apoptosis of leukemia cells, and increased expression of CD47 and HLA-ABC (Supplemental Fig. 5B, C).

Among the 13 patients who received TT, 11 were clinically non-responders. We performed proteomic analysis to assess whether TT reduced leukemia burden, quantified the magnitude of numerical alterations in circulating and BM leukemia cells across patients, and delineate shifts and alterations in the leukemia proteomic landscape under therapy-induced cellular stress (Fig. 1E). The leukemic cell burden was determined before therapy and at D8 or at the earliest available time-point after therapy. Seven patients had substantial ( ≥ 90%) or moderate ( ≥ 50% and <90%) reduction in blast counts, while four patients had mild or no reduction ( ≤ 50%) (Supplemental Fig. 6A). Patients with mild or no reduction in blast fractions tended to cluster together, and those with substantial or moderate reduction in blast fractions also grouped together (Fig. 1F). CD8 + T-cells and NK-cells were significantly enriched in responders, and the myeloid/CD8 + T-cell and myeloid/NK-cell ratios were significantly lower in patients achieving substantial blast reduction after TT (Supplementary Figs. 2H and 6B). TT disrupted the balance between pro- and anti-apoptotic pathways by downregulating MCL1 and YTHDF2, thereby promoting cell death (Fig. 1I, J). Proteomic alteration patterns varied across patients, and upregulation of BCL2, and BCL-xL illustrated potential mechanisms of survival under therapy stress (Fig. 1I, J).

## Discussion

In this phase I trial of milademetan in combination with LDAC, ±venetoclax for AML, the MTD was determined to be 260 mg/day of milademetan (D 5–7 and D 15–17), 600 mg of venetoclax (D 1–14), and 20 mg of LDAC administered twice daily (D1-10). The treatment combination was associated with noticeable rates of gastrointestinal toxicity (50% patients ≥ grade 3) and only modest clinical responses. Noticeable gastrointestinal toxicity has been also seen in trials with other MDM2i in AML and could be an important adverse class effect of these drugs [6, 9, 14].

Combining BCL2 inhibition with MDM2 inhibition potentiates dual apoptotic pathways [7] and translated into clinical efficacy with idasanutlin and venetoclax combination in a phase Ib trial [8, 15]. However, it did not translate into meaningful clinical responses in our study. One challenge may be that 93% of our patients had prior venetoclax exposure. The phase 3 MIRROS trial that evaluated idasanutlin or placebo added to cytarabine in patients with R/R AML, failed to show any improvement in survival in the 232 patients with *TP53*^*wt*^ treated with cytarabine + idasanutlin [14]. Patients on the idasanutlin arm had increases in *TP53* transcriptional targets highlighting the upregulation of *TP53*^*wt*^ expression. Studies have shown that compensatory increase in BCL-XL and MDM4 levels can drive resistance after therapeutic p53-MDM2 axis perturbations [16]. This could mean that mere inhibition of MDM2 in AML cells might not lead to lasting responses.

MDM2i in myeloid malignancies harbors the possibility of a clonal selection of *TP53* mutated cells; 30% of patients treated with idasanutlin expanded p53 mutated clones after therapy for R/R AML [9]. In that study, almost all clones pre-existed as showed by digital droplet polymerase chain reaction of mutated *TP53* [9]. In our cohort, amongst the 8 patients who had sequential p53 protein expression measured by IHC in the leukemic blasts, only 1 patient went on to develop mutated p53 protein expression and low VAF mutated *TP53*.

Through comprehensive CyTOF analysis we did not detect a substantial or persistent accumulation of p53 in samples assessed at the EOC1 in patients receiving DT, indicating p53 accumulation in response to MDM2i and LDAC is transient which could partly explain the low efficacy of DT. We found that DT downregulated MCL1 levels, which is regulated by p53 through transcriptional regulation and post-translational modification [7]. Previously, we reported that p53 activation through MDM2 inhibition induced MCL1 downregulation through regulation of MCL1 phosphorylation [7] and that reduced MCL1 levels could sensitize leukemic cells to apoptosis induction and synergize with BCL2 inhibitors (BCL2i). In this context, addition of venetoclax to milademetan and LDAC induced substantial cell death by targeting distinct survival pathways. Interestingly, and not previously reported, the abundance of CD8 + T-cells and NK-cell were associated with response to TT. These findings agree with recent preclinical reports demonstrating that anti-tumor effect of both BCL2i and MDM2i is partly mediated by immune cells [17, 18]. Our study patients were heavily pretreated and the previous therapy lines for AML treatment deplete and negatively affect immune cell fitness. Thus, assessment of fitness of the immune compartment could provide clues to predict therapeutic efficacy and inform future clinical trial designs.

Our analysis showed that TT consistently downregulates MCL1 and revealed YTHDF2 downregulation as a novel mechanism of cell death induction in AML. This mechanism could further synergize with MCL1 downregulation and BCL2 inhibition. However, leukemic cells may utilize BCL2 and BCL-XL upregulation as potential resistance mechanisms. Overall, our single-cell proteomic analysis of leukemia and immune cell compartments provides insights into mechanisms of action and treatment resistance in AML, which could guide the development of future drug combinations.

In conclusion, despite the biological rationale behind the dual inhibition of MDM2 and BCL2 to optimize leukemic cell apoptosis and a promising clinical trial the benefit from this approach was not apparent in the present clinical setting. Our phase 1 trial captured noticeable GI toxicities. In-depth correlative CyTOF analysis revealed that leukemic cell death by the combination therapy was mediated by the downregulation of MCL1 and YTHDF2 and favored by an immune-rich microenvironment.

## Supplementary information

Supplemental Methods File

Supplemental Data File

Clinical Trial Protocol

Reproducibility checklist

## Data Availability

Data available from corresponding authors on request
