# Supplementary material for: A Phase I study of Milademetan (DS3032b) in combination with low dose cytarabine with or without venetoclax in acute myeloid leukemia: Clinical safety, efficacy, and correlative analysis
Source: Blood Cancer J. 2023 Jun 29;13(1):101. doi: 10.1038/s41408-023-00871-1 (PMC10310786; doi:10.1038/s41408-023-00871-1)
Supplement: Supplementary file 1 — Supplemental Methods File [file 41408_2023_871_MOESM1_ESM.docx]

**Supplemental Methods File**

**A Phase I study of Milademetan (DS3032b) in combination with low dose cytarabine with or without venetoclax in acute myeloid leukemia: Clinical safety, efficacy, and correlative analysis**

*Jayastu Senapati^1^, *Muharrem Muftuoglu^1^, Jo Ishizawa^1^, Hussein A. Abbas^1^, Sanam Loghavi^2^, Gautam Borthakur^1^, Musa Yilmaz^1^, Ghayas C. Issa^1^, Samuel I.Dara^1^, Mahesh Basyal^1^, Li Li^1^, Kiran Naqvi^1^, Rasoul Pourebrahim^1^, Elias J. Jabbour^1^, Steven M. Kornblau^1^, Nicholas J. Short^1^, Naveen Pemmaraju^1^, Guillermo Garcia-Manero^1^, Farhad Ravandi^1^, Joseph Khoury^2^, Naval Daver^1^, Hagop M. Kantarjian^1^, ^#^Michael Andreeff^1^, ^#^Courtney D. DiNardo^1^

*^1^Department of Leukemia, MD Anderson Cancer Center, Houston, Texas*

*^2^Department of Hematopathology, MD Anderson Cancer Center, Houston, Texas*

*JS and MM are co-first authors and contributed equally

^#^ MA and CD are co-senior authors and contributed equally

**Pages-** 6

**Tables-** 1

**CyTOF METHODS**

**Samples**

Serial peripheral blood (PB) and (BM) samples were collected from patients enrolled in Milademetan + low dose cytarabine +/- venetoclax trial (NCT03634228) and informed consents were obtained in accordance with The University of Texas MD Anderson Cancer Center Institutional Review Board (IRB) guidelines. Mononuclear cells from PB and BM samples were isolated as described previously[1] by density-gradient centrifugation using Lymphoprep separation medium (Axis Shield, Oslo, Norway). Mononuclear cells were frozen down in cell freezing media containing 90% fetal bovine serum and 10% dimethyl sulfoxide (DMSO). Frozen samples were stored in liquid nitrogen until further use.

Serially collected patient PB and BM samples were thawed in a single batch and processed simultaneously. Frozen cells were thawed in 37^o^ C water bath and then transferred to pre-warmed cell thawing media containing 80% RPMI 1640, 20 % fetal bovine serum, 50 IU-ml benzonase (Sigma-Aldrich, St. Louis, MO). Cells were washed twice, resuspended in 80% RPMI 1640 and 20 % fetal bovine serum, plated in 96-well plate at desired concentration and incubated at 37° C with air containing 5% CO_2_ up to 6h prior to staining.

**Antibodies**

A 51-paremater, in-house developed, leukemia-focused CyTOF panel was utilized and antibodies and corresponding metals are detailed in Table S1. Carrier-free, unlabeled antibodies were conjugated to lanthanides, indium isotopes (113In and 115In) and bismuth isotope (209Bi) using Maxpar X8 antibody conjugation kit per manufacturer’s instructions (Standard Bio Tools, San Francisco, CA). Lanthanides, indium and bismuth isotopes were procured from Standard Bio Tools, Traces Sciences and Sigma-Aldrich, respectively. Monoisotopic Indium salts were dissolved in distilled H2O to 1 M stock solution, then further diluted to 50 mM in L buffer (Standard Bio Tools, San Francisco, CA) and loaded onto Maxpar X8 polymer (Standard Bio Tools, San Francisco, CA) as described previously[1]. Bismuth was dissolved in 5% nitric acid at a 50mM concentration[2]. Four monoisotopic cisplatin compounds (194Pt, 195Pt, 196Pt, 198Pt), dissolved at 5mM in DMSO, were purchased from Standard Bio Tools and used for antibody conjugation. Antibody tagging to monoisotopic cisplatin was performed as described previously[1, 3]. For conjugation, we used 100-200 µg of unlabeled carrier-free antibodies. Following a single wash using R buffer in a 50 kilodalton (kDa) spin filter column (EMD Millipore), antibodies were reduced in R buffer using 4 mM final concentration of TCEP for 30 min at 37^o^ C and then washed twice with C buffer. The reduced antibody was resuspended in C-buffer containing metal-loaded X8 polymers. Monoisotopic cisplatin compounds containing either 194Pt, 195Pt or 198Pt (Standard Bio Tools, San Francisco, CA) was added to reduced antibodies in a volume of 400 µl C buffer at a final concentration of 100 µM. The antibody and metal-loaded polymer or monoisotopic cisplatin mixture was incubated for two hours at 37^o^ C and washed four times with W buffer (Fluidigm, San Francisco, CA). Antibody concentration was determined by absorbance reading 280 nm using Nanodrop 2000 (Thermo Fisher Scientific, Waltham, MA) All conjugated antibodies were then diluted to 0.5 mg/ml final concentration in PBS-based antibody stabilization solution (Candor Bioscience GmbH, Wangen, Germany) supplemented with 0.05% sodium azide (Sigma-Aldrich, St. Louis, MO). Serial titration experiments were performed to determine the concentration giving the optimal signal-to-noise ratio for each antibody.

Antibody conjugation to cadmium (Cd) isotopes, 111Cd, 112Cd, 114Cd and 116Cd, was performed as described previously[1]. In brief, 13 µl of Cd isotopes (Standard Bio Tools, San Francisco, CA) were loaded onto 200 µg of MCP polymer suspended in 87 µl of L buffer and incubated for 1h at 37 °C in water bath. Cd isotopes were then loaded to MCP9 polymer, washed twice in L and once in C buffer on 3 kDa spin filter and finally suspended in 60 µl C-buffer per manufacturer’s instructions. 100 µg of antibodies was washed twice in R buffer and then reduced in 4 mM TCEP in 100 µl R buffer for 30 min at 37 °C. The reduced antibody was then washed twice in C-buffer. Cd-loaded MCP polymer was then transferred to the 50 kDa filter containing the reduced antibody. Reduced antibody/Cd-loaded polymer mixture was incubated for 90 min at 37 °C. After incubation, antibodies were transferred to 100 kDa filter and washed four times for five min at 5000 × *g*. Antibody concentration was determined based on absorption at 280 nm using Nanodrop 2000 (Thermo Fisher Scientific, Waltham, MA) All conjugated antibodies were then diluted to 0.5 mg/ml final concentration in HRP-protector (Candor Bioscience GmbH, Wangen, Germany)

**Sample Barcoding**

Samples were barcoded using Cell-ID 20-Plex Pd barcoding kit (Standard Bio Tools, San Francisco, CA), which utilized triple combinations of 6 different palladium (Pd) isotopes. Cells were fixed, washed once in 1X barcode perm buffer, and then resuspended in 800 μL barcode perm buffer. Barcodes were thawed at RT, quick-spun and resuspended in 100 μL barcode perm buffer. Barcodes were transferred to appropriate samples, mixed thoroughly and incubated at RT for 30 min. Samples then washed 3X in cell staining buffer (0.5% bovine serum albmin (BSA) in PBS). Cells were resuspended in 100 μL PBS, transferred and collected in a single tube. Cells were counted prior to staining, spun down and resuspended in cell staining buffer.

**IdU labeling and sample staining**

Following up to 6h culture at 37^o^C in humidified air containing 5% CO_2_ cells were labeled with 5-Iodo-2’-deoxyuridine (IdU) (Acros Organics) to mark cells in S-phase of cell cycle. Cells were incubated at a final concentration of 10 μM IdU for 30’ at 37^o^C/5% CO_2_ and then washed 2X in cell staining buffer. Cell were resuspended in cell staining buffer, Fc blocked for 10’ with Human TruStain FcX™ solution (Biolegend) and then stained with a mixture of surface antibodies (**Table A**) in a final volume of 100 µl per 3 million cells. Samples were incubated for 30 min RT. Cisplatin (Enzo) was added at 2uM final concentration to exclude dead cells 2 min prior to sample wash. Cells were wash 2X in cell staining buffer and then fixed in 500 µl of 1.6%, diluted in PBS, and spun down at 2000 rpm for 5 min. To permeabilize the samples, cells were suspended in 100 μl cell staining buffer and 900 μl ice-cold Methanol was added. After 60 min of incubation at -20, cells were washed twice with staining buffer and stained with intracellular antibodies for 30 min at 4^o^C. Following intracellular staining, cells were washed twice and resuspended in intercalator solution (1.6% PFA in PBS with 125 nM iridium nucleic acid intercalator) and incubated at 4 °C overnight.

**Sample acquisition and data processing**

Cells labeled with Cell-ID iridium intercalator (Standard Bio Tools, San Francisco, CA) overnight were washed twice in cell staining buffer, resuspended in 1 ml of MilliQ dH_2_O, filtered using cell strainer cap tubes with 35 µm nylon mesh (BD Bioscience, San Jose, CA), counted and spun down before sample introduction. Samples were suspended at a concentration of 0.6 × 106/ml in MilliQ ddH_2_O supplemented with 10% EQ Four Element Calibration Beads (Standard Bio Tools, San Francisco, CA) and acquired at 300-500 events per second on Helios instrument using CyTOF Software version 6.7.1016 (Standard Bio Tools, San Francisco, CA). CyTOF data were normalized based on signal drift over time using CyTOF Software.

**Data Analysis**

Barcoded sample deconvolution was performed with the debarcoder software (Standard Bio Tools, San Francisco, CA) or Premessa R-package. (<https://github.com/ParkerICI/premessa>). De-barcoding parameters were set to achieve optimal barcode separation. Data processing and clean-up of deconvoluted samples was performed by using Flowjo version 10.8.1. Calibration beads were gated out and singlets were chosen based upon DNA content and event length. Dead cells were excluded by selecting cells with low cisplatin uptake. Desired populations of interest were gated on and then exported for downstream analyses. We used t-Distributed Stochastic Neighbor Embedding (t-SNE)[4] and uniform manifold approximation and projection (UMAP)[5] for dimension reduction, and FlowSOM[6] and PhenoGraph clustering algorithms in Cytofkit[7] , Omiq.ai platform and Flowjo[8] (BD Bioscience, San Jose, CA). Principal component analysis and differential expression analysis were performed using Seurat package[9] in R environment. Single-cell expression heatmaps were generated using ComplexHeatmap package[10] to display arcsinh-transformed expression levels across indicated number of cells.

**Statistical Analysis**

Statistical analyses were performed using Prism software version 9.0 (GraphPad Software Inc.) and R version 4.0.1. The statistical differences between matched groups were compared using a paired *t* test using GraphPad Software. To compare unpaired groups and for differential expression analysis, we used Mann-Whitney *U* test. The Spearman rank correlation was used to assess correlation. (Statistical significance was set at *P* < 0.05: **P* < 0.05; ***P* < 0.01; ****P* < 0.001; *****P* < 0.0001.)

**Table A: Index of surface antibodies**

| **Isotope** | **Metal** | **Antigen** | **Clone** | **Vendor** | **Category** |
| --- | --- | --- | --- | --- | --- |
| 89 | Y | CD45 | HI30 | Standard Bio Tools | Surface |
| 102 | Pd | N/A | N/A | Standard Bio Tools | Barcode |
| 104 | Pd | N/A | N/A | Standard Bio Tools | Barcode |
| 105 | Pd | N/A | N/A | Standard Bio Tools | Barcode |
| 106 | Pd | N/A | N/A | Standard Bio Tools | Barcode |
| 108 | Pd | N/A | N/A | Standard Bio Tools | Barcode |
| 110 | Pd | N/A | N/A | Standard Bio Tools | Barcode |
| 111 | Cd | CD11b | ICRF44 | Biolegend | Surface |
| 112 | Cd | CLA | HECA-452 | BD Biosciences | Surface |
| 113 | In | CD8 | RPA-T8 | Biolegend | Surface |
| 114 | Cd | CD47 | CC2C6 | Biolegend | Surface |
| 115 | In | CD3 | UCTH1 | Biolegend | Surface |
| 116 | Cd | γH2AX | 2F3 | Biolegend | Intra |
| 127 | I | N/A | N/A | Acros | Intra |
| 139 | La | CD36 | 5-271 | Biolegend | Surface |
| 140 | Ce | Ubiquitin | EPR8830 | Abcam | Intra |
| 141 | Pr | Bcl-xL | 54H6 | Cell Signaling | Intra |
| 142 | Nd | CD68 | KP1 | ThermoFisher | Intra |
| 143 | Nd | CD56 | NCAM16.2 | BD Biosciences | Surface |
| 144 | Nd | Bcl-2 | 100 | Biolegend | Intra |
| 145 | Nd | CD123 | 6H6 | Biolegend | Surface |
| 146 | Nd | BIM | C34C5 | Cell Signaling | Intra |
| 147 | Sm | p-4EBP1 | 236B4 | Cell Signaling | Intra |
| 148 | Nd | CD34 | 581 | Biolegend | Surface |
| 149 | Sm | Bad | Y208 | Abcam | Intra |
| 150 | Nd | p-STAT5 | 47 | BD Biosciences | Intra |
| 151 | Eu | CLL1 | 50C1 | Biolegend | Surface |
| 152 | Sm | p-STAT3 | 4/P-STAT3 | BD Biosciences | Intra |
| 153 | Eu | p21 | 12D1 | Cell Signaling | Intra |
| 154 | Sm | Ki-67 | 2raj1 | ThermoFisher | Intra |
| 155 | Gd | PDL1 | 29E.2A3 | Biolegend | Surface |
| 156 | Gd | CD38 | HB-7 | Biolegend | Surface |
| 157 | Gd | CD19 | HIB19 | Biolegend | Surface |
| 158 | Gd | CD33 | WM53 | Biolegend | Surface |
| 159 | Gd | p-AKT | M89-61 | BD Biosciences | Intra |
| 160 | Dy | YTHDF2 | Polyclonal | Proteintech | Intra |
| 161 | Dy | p-GSK3 | Polyclonal | R&D | Intra |
| 162 | Dy | Survivin | Polyclonal | R&D | Intra |
| 163 | Dy | c-myc | D84C12 | Cell Signaling | Intra |
| 164 | Dy | MDM2 | D-12 | SantaCruz | Intra |
| 165 | Ho | p53 | DO-7 | BD Biosciences | Intra |
| 166 | Er | c-kit | 104D2 | Biolegend | Surface |
| 167 | Er | p-ERK1/2, p44/42 MAPK | D13.14.4.E | Cell Signaling | Intra |
| 168 | Er | Noxa | 114C307.1 | Abcam | Intra |
| 169 | Tm | p-MEK1/2 | 41G9 | Cell Signaling | Intra |
| 170 | Er | PUMA | EP512Y | Abcam | Intra |
| 171 | Yb | CD4 | RPA-T4 | Biolegend | Surface |
| 172 | Yb | p-S6 | D68F8 | Cell Signaling | Intra |
| 173 | Yb | Bax | 2D2 | Biolegend | Intra |
| 174 | Yb | p-FLT3 | 30D4 | Cell Signaling | Intra |
| 175 | Lu | CXCR4 | 12G5 | BD Biosciences | Surface |
| 176 | Yb | Mcl1 | D2W9E | Cell Signaling | Intra |
| 191 | Ir | N/A | N/A | Standard Bio Tools | Intra |
| 193 | Ir | N/A | N/A | Standard Bio Tools | Intra |
| 194 | Pt | CD15 | W6D3 | Dead cells | Surface |
| 195 | Pt | HLA-ABC | W6/32 | Biolegend | Surface |
| 196 | Pt | Live-dead | N/A | Enzo | N/A |
| 198 | Pt | HLA-DR | L243 | Biolegend | Surface |
| 209 | Bi | H3K27 | C36B11 | Cell Signaling | Intra |

**References**

1. Muftuoglu, M., et al., *Extended live-cell barcoding approach for multiplexed mass cytometry.* Scientific reports, 2021. **11**(1): p. 1-13.

2. Han, G., et al., *Atomic mass tag of bismuth-209 for increasing the immunoassay multiplexing capacity of mass cytometry.* Cytometry A, 2017. **91**(12): p. 1150-1163.

3. Mei, H.E., M.D. Leipold, and H.T. Maecker, *Platinum-conjugated antibodies for application in mass cytometry.* Cytometry A, 2016. **89**(3): p. 292-300.

4. Amir el, A.D., et al., *viSNE enables visualization of high dimensional single-cell data and reveals phenotypic heterogeneity of leukemia.* Nat Biotechnol, 2013. **31**(6): p. 545-52.

5. Becht, E., et al., *Dimensionality reduction for visualizing single-cell data using UMAP.* Nat Biotechnol, 2018.

6. Van Gassen, S., et al., *FlowSOM: Using self-organizing maps for visualization and interpretation of cytometry data.* Cytometry A, 2015. **87**(7): p. 636-45.

7. Chen, H., et al., *Cytofkit: A Bioconductor Package for an Integrated Mass Cytometry Data Analysis Pipeline.* PLoS Comput Biol, 2016. **12**(9): p. e1005112.

8. Li, L., et al., *In-depth analysis of SARS-CoV-2-specific T cells reveals diverse differentiation hierarchies in vaccinated individuals.* JCI Insight, 2022. **7**(7).

9. Butler, A., et al., *Integrating single-cell transcriptomic data across different conditions, technologies, and species.* Nat Biotechnol, 2018. **36**(5): p. 411-420.

10. Gu, Z., R. Eils, and M. Schlesner, *Complex heatmaps reveal patterns and correlations in multidimensional genomic data.* Bioinformatics, 2016. **32**(18): p. 2847-9.
