# Supplementary material for: A Phase I study of Milademetan (DS3032b) in combination with low dose cytarabine with or without venetoclax in acute myeloid leukemia: Clinical safety, efficacy, and correlative analysis
Source: Blood Cancer J. 2023 Jun 29;13(1):101. doi: 10.1038/s41408-023-00871-1 (PMC10310786; doi:10.1038/s41408-023-00871-1)
Supplement: Supplementary file 2 — Supplemental Data File [file 41408_2023_871_MOESM2_ESM.docx]

**Supplemental data file: Tables and Figures**

**A Phase I study of Milademetan (DS3032b) in combination with low dose cytarabine with or without venetoclax in acute myeloid leukemia: Clinical safety, efficacy, and correlative analysis**

*Jayastu Senapati^1^, *Muharrem Muftuoglu^1^, Jo Ishizawa^1^, Hussein A. Abbas^1^, Sanam Loghavi^2^, Gautam Borthakur^1^, Musa Yilmaz^1^, Ghayas C. Issa^1^, Samuel I.Dara^1^, Mahesh Basyal^1^, Li Li^1^, Kiran Naqvi^1^ Rasoul Pourebrahim^1^, Elias J. Jabbour^1^, Steven M. Kornblau^1^, Nicholas J. Short^1^, Naveen Pemmaraju^1^, Guillermo Garcia-Manero^1^, Farhad Ravandi^1^, Joseph Khoury^2^, Naval Daver^1^, ^#^Michael Andreeff^1^, ^#^Courtney DiNardo^1^

*^1^Department of Leukemia, MD Anderson Cancer Center, Houston, Texas*

*^2^Department of Hematopathology, MD Anderson Cancer Center, Houston, Texas*

*JS and MM are co-first authors and contributed equally

^#^ MA and CD are co-senior authors and contributed equally

**Pages- 9**

**Tables- 1**

**Figures- 6**

**Figure 1: Phase 1 treatment protocol and drug dosage**


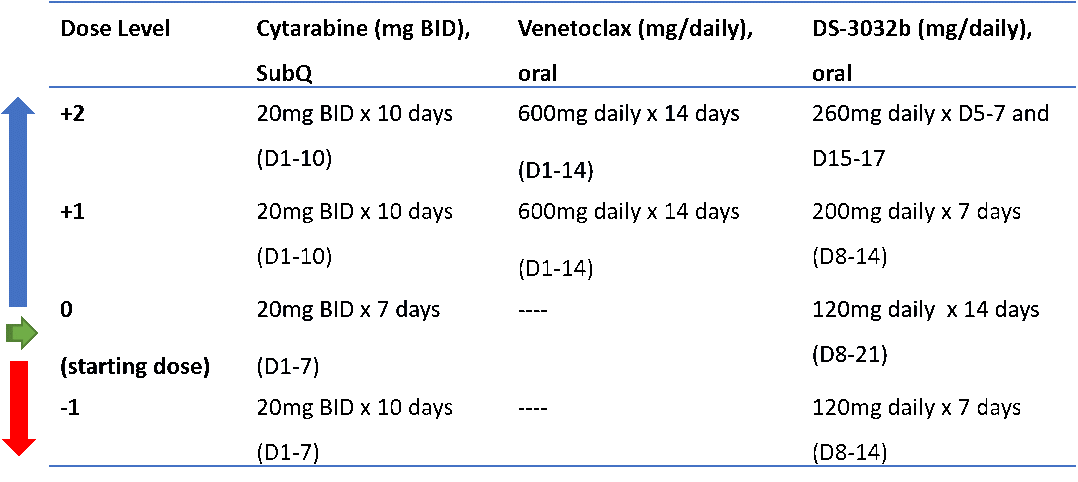


**Abbreviations:** BID, twice daily; SubQ, subcutaneous; D, day

**Figure 2:** Oncoprint of study patients at trial therapy initiation


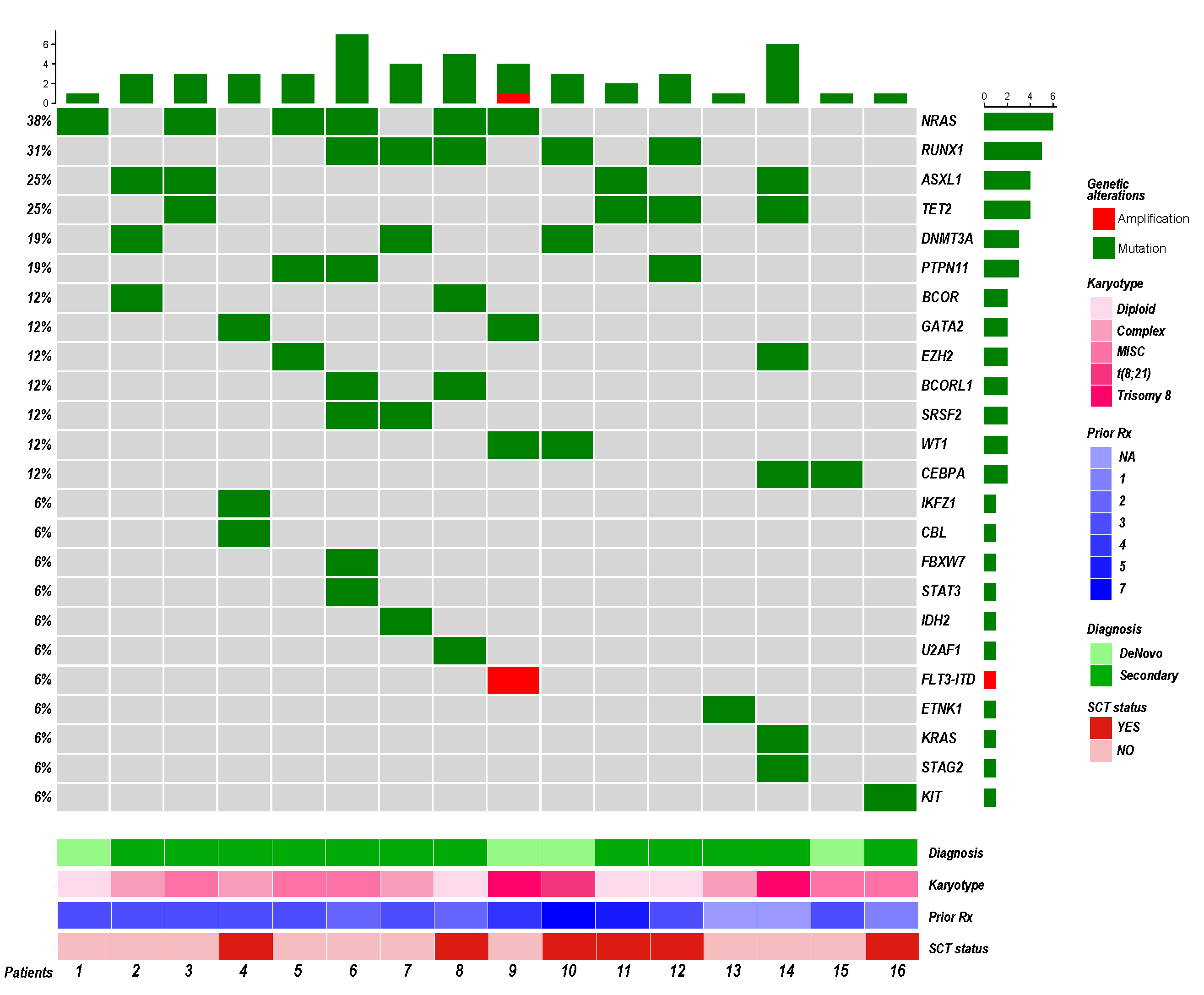


**Abbreviations**: MISC, miscellaneous; SCT, stem cell transplantation; Rx, therapy

**Table 1:** Treatment emergent adverse effects

| Adverse events | | All grades | Grade 3 | Grade 4 |
| --- | --- | --- | --- | --- |
| Gastrointestinal | Diarrhea  Infectious enterocolitis  Proctitis  Ileus | 7 (44)  4 (25)  1 (6)  1 (6) | 1 (6)  4 (25)  1 (6)  1 (6) | 1 (6)  0 (0)  0 (0)  0 (0) |
| Infections  (non-Gastro-intestinal) | Lung infection  Sepsis  Hepatic infection  Skin infection  Salivary gland infection | 10 (63)  6 (37)  1 (6)  1 (6)  1 (6) | 10 (63)  5 (31)  1 (6)  1 (6)  1 (6) | 0 (0)  1 (6)  0 (0)  0 (0)  0 (0) |
| Miscellaneous | Elevated creatinine  Encephalopathy  Multi-organ failure  Hypoxia  Seizures  Hypertension  Non-cardiac chest pain | 3 (19)  1 (6)  1 (6)  1 (6)  1 (6)  1 (6)  1 (6) | 2 (13)  1 (6)  0 (0)  1 (6)  1 (6)  1 (6)  0 (0) | 0 (0)  1 (6)  0 (0)  0 (0)  0 (0)  0 (0)  0 (0) |

**Figure 3:** Indications for study discontinuation and overall patient disposition


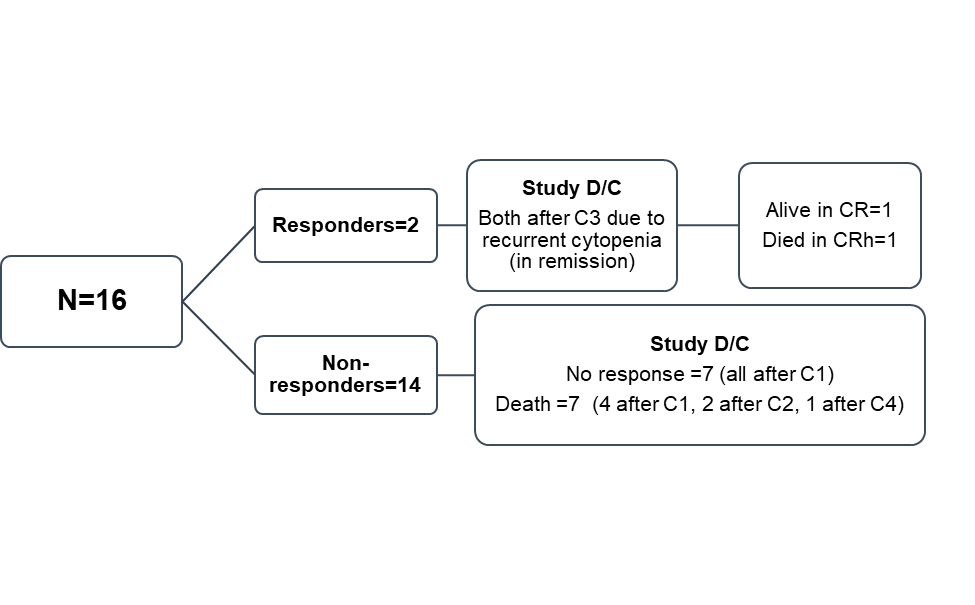


**Figure 4: CyTOF analysis of proteomic landscape in patients received doublet therapy**.


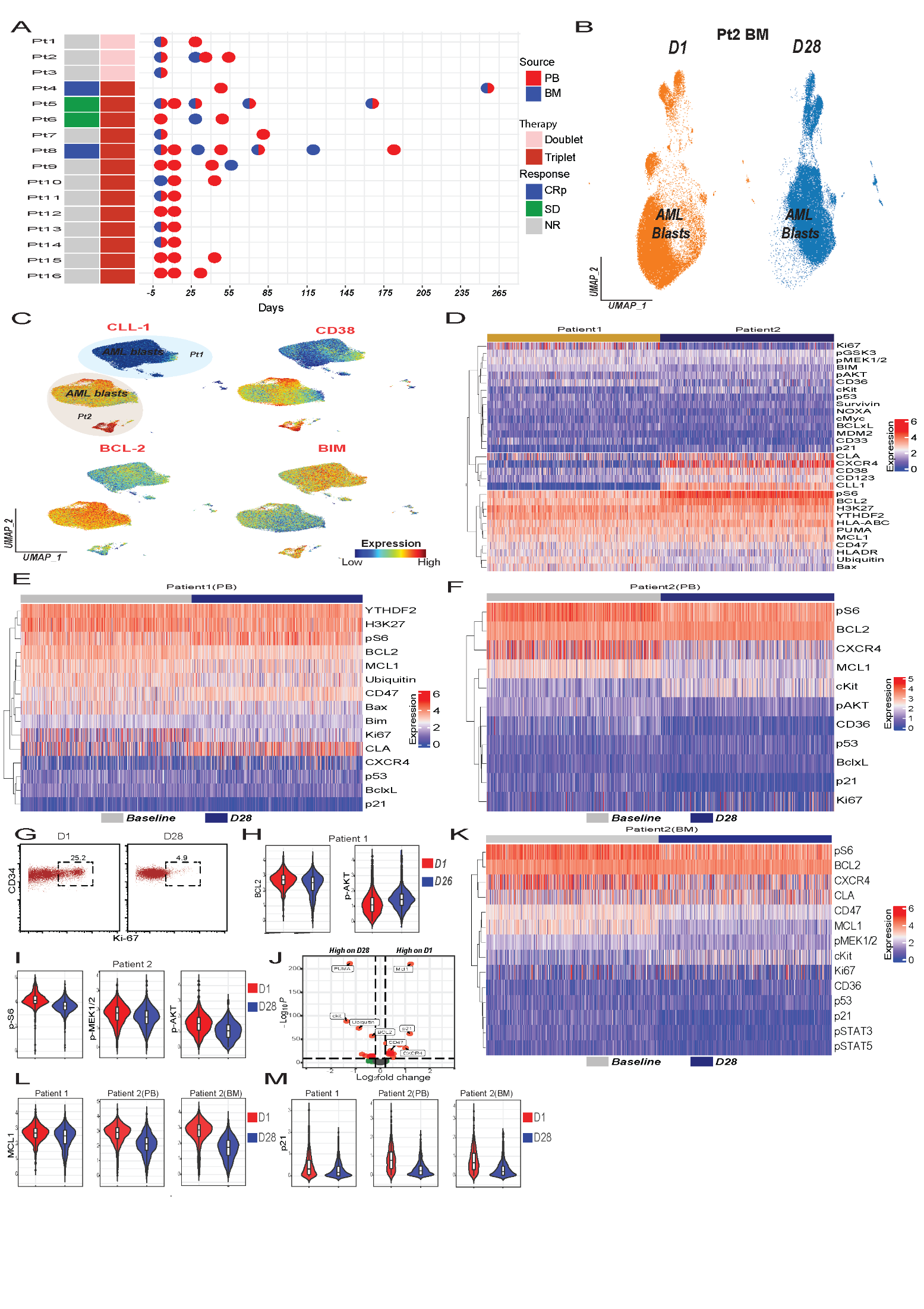


**CyTOF analysis of proteomic landscape in patients received doublet therapy**. **A)** Study scheme summarizing patient samples analyzed using CyTOF, clinical response, sample source and therapy regimen. Scatterpie plot displays sample collection timepoints represented by circles. Red and blue colors denote peripheral blood and bone marrow samples, respectively. **B)**  UMAP plots of pre- and post-treatment BM samples of Pt2 (upper panel). **C)** UMAP plots of pre- and post-treatment PB samples collected from patients, Pt1 and Pt2, are colored for the indicated markers. **D)** Single-cell protein expression heatmap showing expression of the indicated markers (rows) across Pt1 and Pt2 PB baseline leukemia cells. Color bar indicates arcsinh-transformed marker expression levels. **E)** Single-cell protein expression heatmap showing expression of the indicated markers (rows) across D1 and D28 PB leukemia cells from Pt1. Color bar indicates arcsinh-transformed marker expression levels. **F)** Single-cell protein expression heatmap showing expression of the indicated markers (rows) across D1 and D28 PB leukemia cells from Pt2. Color bar indicates arcsinh-transformed marker expression levels. **G)** FACS plots showing the Ki-67 expression of leukemia cells from Pt1 on D1 and D28. **H)** Violin plot showing BCL2 (left) and p-AKT (right) expression in Pt1 leukemia cells on D1 (red) and D28 (blue). **I)** Violin plot shows the expression of p-S6 (left), p-MEK1/2 (middle) and p-AKT (right) in Pt2 leukemia cells on D1 (red) and D28 (blue). **J)** Volcano plot showing the differentially expressed proteins in BM leukemia cells from Pt2 assessed on D1 vs D28. Features shown on the right-hand side are detected at higher levels on D1. The threshold in the volcano plot was -log10 adjusted P>12 and log2 fold change >0.25. **K)** Single-cell protein expression heatmap showing expression of the indicated markers (rows) across D1 and D28 BM leukemia cells from Pt2. Color bar indicates arcsinh-transformed marker expression levels. **L)** Violin plots showing MCL1 expression before (red) and after (blue) therapy in leukemia cells from Pt1 PB (left), Pt2 PB (middle) and BM (right). **M)** Violin plots showing p21 expression before (red) and after (blue) therapy in leukemia cells from Pt1 PB (left), Pt2 PB (middle) and BM (right).


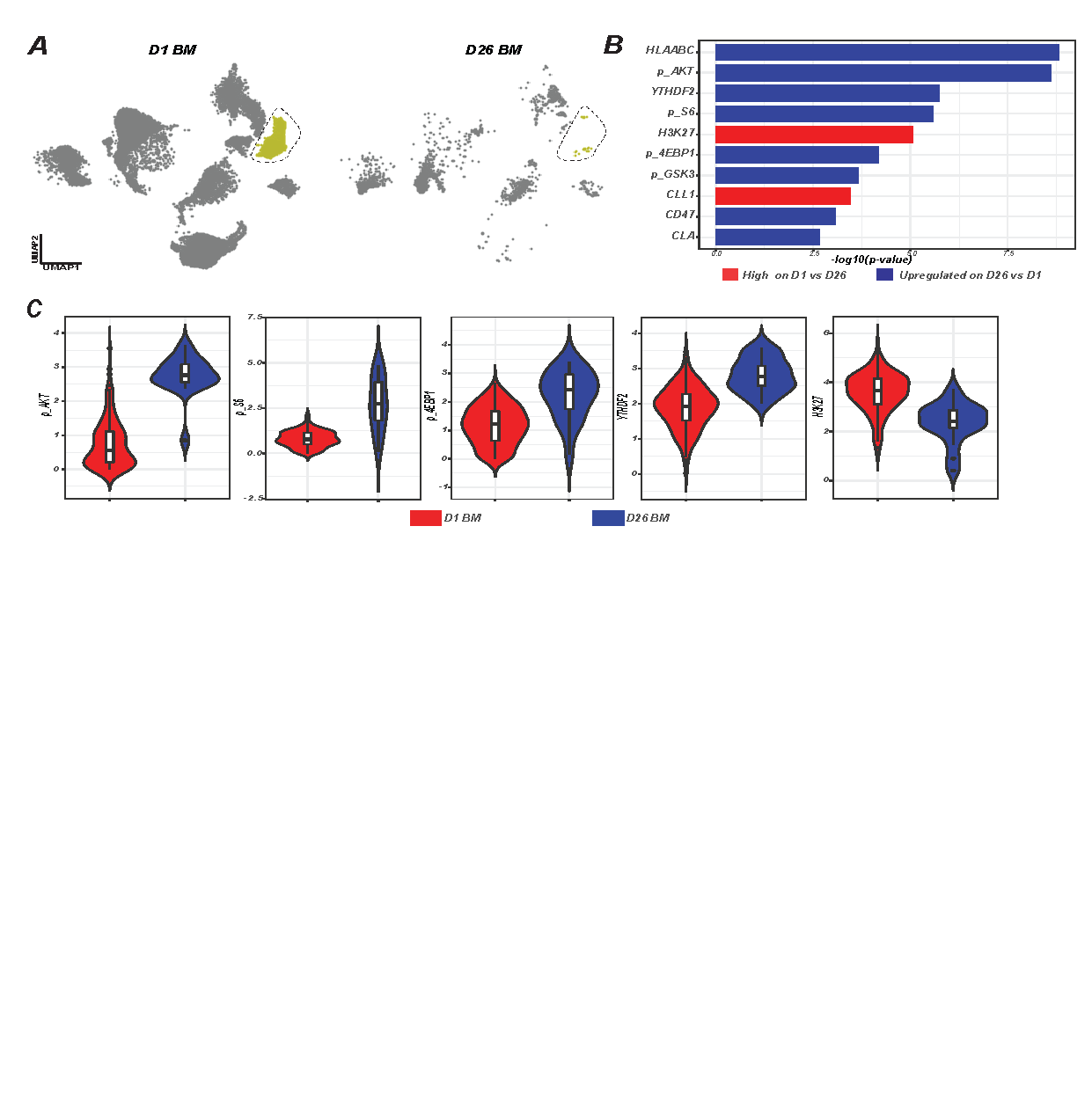
**Figure 5: CyTOF analysis of proteomic landscape in patient achieved CR**.

**CyTOF analysis of proteomic landscape a patient achieved complete remission after triplet therapy**. **A**) D1 and D26 BM samples are selected from the 8 pooled samples shown in Figure 2C and are plotted using the same UMAP coordinates. UMAP plots depict the proteomic landscape of Pt8 on D1 (**left**) and D26 (**right**) BM samples. Leukemia cells are colored in dark yellow and non-malignant hematopoietic cells shown in gray. **B**) Bar chart shows top differentially expressed features in BM leukemia cells on D26 compared to D1 BM leukemia cells. Protein expression levels of leukemia cells shown in **A** are used for DEA. Blue and red colors indicate increase and decrease in expression levels of the indicated markers on D26 compared to D1, respectively. **C**) Violin plot shows expression of the indicated markers in BM leukemia cells shown in **A**. Expression levels of pAKT, pS6, p4EBP1, YTHDF2 and H3K27 on D26 in comparison to D1 are shown.

**Figure 6:** The frequencies of cell subsets in leukemia compartments in non-responders


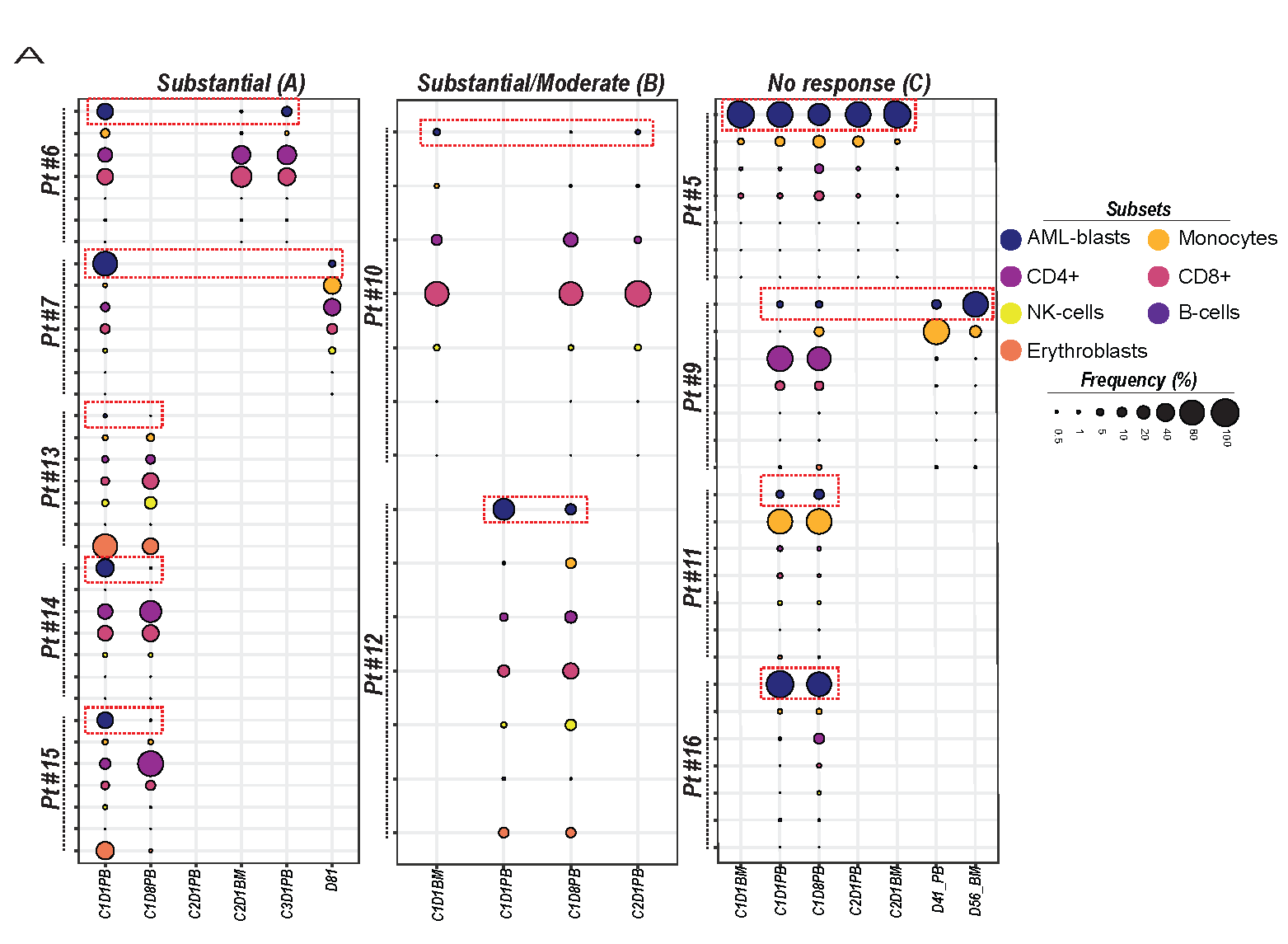


**A**

**B**


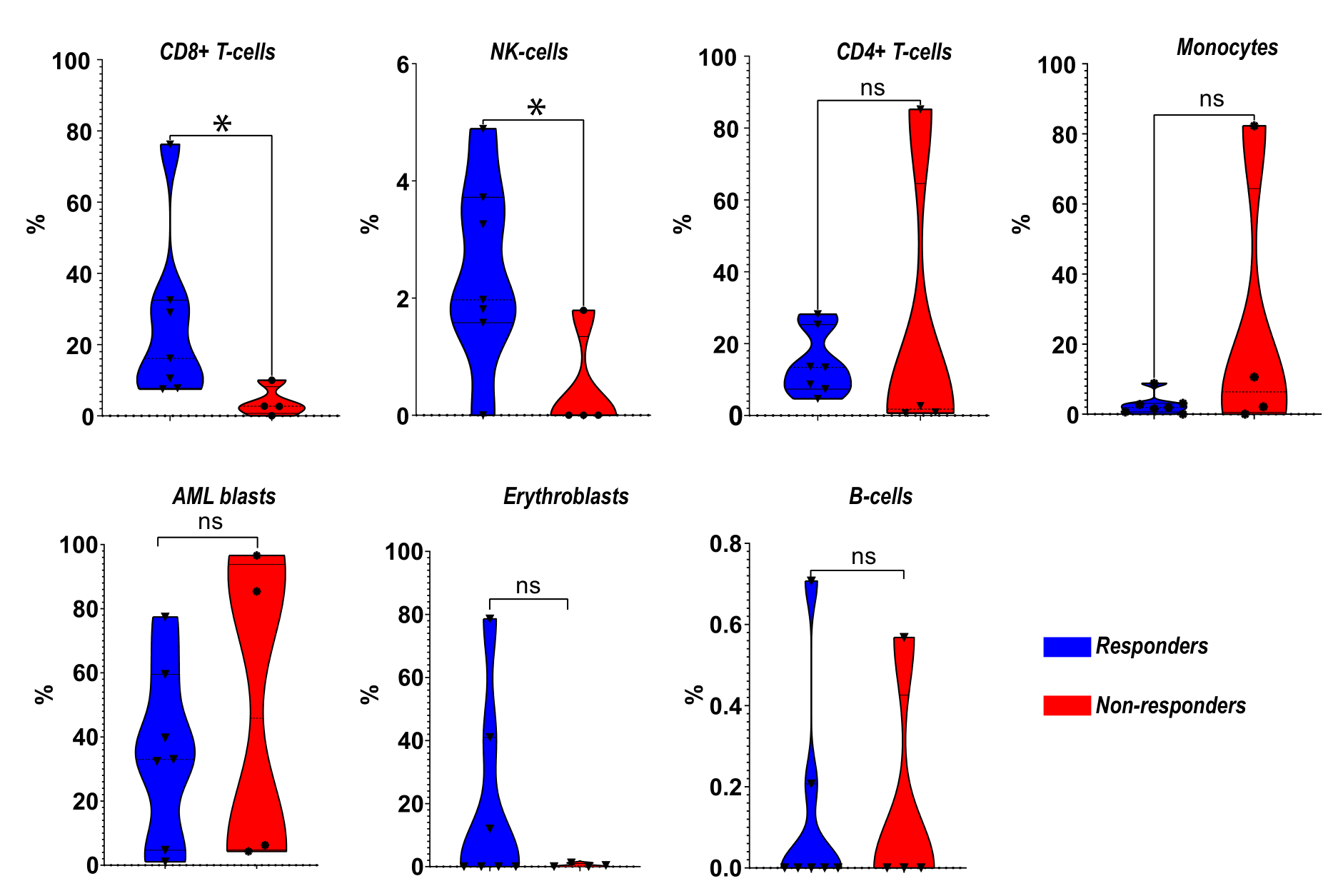


**Temporal Distribution of Subset Frequencies in Leukemia Compartments of Non-Responders Undergoing Triplet Therapy.** **A)** Serial PB and BM samples from eleven non-responders were subjected to UMAP dimension reduction. The frequencies of cell types identified through unsupervised analysis were enumerated. Bubble plots show patient subgroups achieving substantial (≥90%), moderate (≥50% and <90%) and minor or no reduction (≤50%) in blast counts after triplet therapy. Node size indicates cell abundance. Frequencies of leukemia blasts (blue), monocytes (orange), CD4+ T-cells (purple), CD8+ T-cells (dark pink), NK-cells (yellow), B-cells (dark purple) and erythroblasts (dark orange) are shown across indicated time-points. **B)** Violin plots showing baseline frequencies of cell subsets identified through UMAP analysis in responders (patient with significant blast reduction) (blue) and non-responders (patient with mild or no blast reduction, red, n=4). Mann-Whitney test was used for statistical analysis. * indicates p value < 0.05.
