# Supplementary material for: A Phase I study of Milademetan (DS3032b) in combination with low dose cytarabine with or without venetoclax in acute myeloid leukemia: Clinical safety, efficacy, and correlative analysis
Source: Blood Cancer J. 2023 Jun 29;13(1):101. doi: 10.1038/s41408-023-00871-1 (PMC10310786; doi:10.1038/s41408-023-00871-1)
Supplement: Supplementary file 3 — Clinical Trial Protocol [file 41408_2023_871_MOESM3_ESM.pdf]

MD Anderson IND Sponsor Cover Sheet	
**Protocol ID**	2018-0333
**Protocol Title**	A Phase I/II study of the oral MDM2 inhibitor DS-3032b (Milademetan) in combination with low dose cytarabine (LDAC), in patients with newly diagnosed or relapsed/refractory acute myeloid leukemia (AML)
**Protocol Phase**	I/II
**Protocol Version**	03
**Version Date**	08/03/2020
**Protocol PI**	Courtney DiNardo
**Department**	Leukemia
**IND Sponsor**	MD Anderson Cancer Center
**IND #**	140658

Study Title: A Phase I/II study of the oral MDM2 inhibitor DS-3032b (Milademetan) in combination with low dose cytarabine (LDAC), in patients with newly diagnosed or relapsed/refractory acute myeloid leukemia (AML)

Institution Name: MD Anderson Cancer Center

Protocol

PI: Courtney DiNardo, MD
Unit 428
1515 Holcolmbe Blvd
Houston, TX 77030
cdinardo@mdanderson.org
O: 713-794-1141
F: 713-745-4612

TABLE OF CONTENTS

Page

1. **Objectives** 3
2. **Background** 3
3. **Subject Eligibility** 6
4. **Treatment Plan** 8
5. **Study Procedures** 13
6. **Response Definitions** 18
7. **Regulatory and Reporting Requirements** 24
8. **Statistical Considerations** 26

References ....

1. OBJECTIVES

1.1 Primary Objectives

The primary objectives of this study are to evaluate the safety, tolerability and determine the recommended phase 2 dose (Phase 1) and efficacy (by IWG criteria – Phase 2) of the MDM2 inhibitor, DS-3032b, in combination with LDAC, with or without the addition of venetoclax in both the frontline and relapsed/refractory (non-TP53 mutant) AML patient population.

1.2 Secondary Objectives

The secondary objectives of the study are:

1.2.1. Evaluation of time to response variables including overall survival (OS), event-free survival (EFS) and duration of response (DOR)

1.2.2. Determine biomarkers that may be predictive of DS-3032b activity.

1.2.3. Molecular profiling at screening, on study, and at relapse to determine genomic predictors of response and resistance

2. BACKGROUND AND SCIENTIFIC RATIONALE

2.1: Overview of AML:

Outcomes for patients with AML remain poor. Cytarabine has been the mainstay of AML therapy for nearly 40 years. In young patients, cytarabine is usually combined with an anthracycline. With this standard therapy the complete remission (CR) rates are 60-70% and the cure rates are 15-35%. Younger patients (age < 50 years) with diploid karyotype have CR rates of 70-80% and cure rates of 20-25%. Older patients and those with adverse karyotypes have CR rates of 35-50% with cure rates of 10% or less. This is due in part to the poor tolerance to therapy, and also the higher frequency of poor prognostic features, such as high-risk cytogenetic abnormalities and molecular mutations such as mutation of potent tumor suppressor gene - *TP53* [1-3]. Low-dose cytarabine (LDAC) is better tolerated in this

patient population and has been shown to improve survival compared to supportive care ± hydroxyurea, and has therefore become a "de facto" standard therapy for older patients or patients not fit for intensive chemotherapy. However, the median survival is still only approximately 4 months. Thus, there is a need to improve the results obtained with standard chemotherapy. Efforts to improve both the remission rate and the durability of remission in AML patients of all ages are paramount. The tumor suppressor gene *TP53* plays a central role in the regulation of cell cycle, apoptosis, DNA repair and senescence [4]. Murine double minute 2 (MDM2) is an "oncoprotein" that functions by inhibiting wild-type TP53 and thereby resulting in tumorigenesis [5]. Disruption of this MDM2-TP53 interaction is thus an important therapeutic target in cancers retaining wild-type TP53 [6-8]. Previously, MDM2 inhibitors (like Nutlin-3 and MI-63) have shown activity in hematological malignancies, including AML and CLL. DS-3032b, a potent and small molecule inhibitor of MDM2 has demonstrated anti-tumor activity in preclinical studies and has also shown clinical benefit in solid tumors [9]. More recently in a phase 1 study, DS-3032b demonstrated clinical activity as a single agent in relapsed/refractory AML and high-risk MDS in wild-type *TP53* patients [10].

Additionally, many hematologic malignancies are highly dependent upon the anti-apoptotic protein BCL-2 for survival. Over-expression of BCL-2 is associated with tumor initiation, disease progression, and drug resistance, and is therefore a compelling target for antitumor therapy. Venetoclax is a potent, selective and orally bioavailable small-molecule inhibitor of BCL-2 that binds with > 1,000-fold higher affinity for BCL-2 (Ki < 0.010 nM) than for BCL-XL (Ki = 48 nm) or MCL-1 (Ki > 444 nM) [11]. In vitro, venetoclax has demonstrated cell killing activity against patient-derived chronic lymphocytic leukemia (CLL) cells and a variety of lymphoma and leukemia cell lines, including acute myeloid leukemia (AML) [11]. As a single agent for patients with R/R AML, venetoclax has demonstrated clinical activity although responses were relatively modest and short-lived . Synergistic activity against myeloid malignancies is seen in vitro and in vivo with venetoclax in combination with lower intensity antileukemia therapy, such as low-dose cytarabine (LDAC) or hypomethylatingagents (HMA) (i.e., azacitidine or decitabine) [13-16].Clinical data are now also available demonstrating encouraging safety and efficacy in treatment-naive elderly and/or unfit AML patients, with venetoclax in combination with either LDAC (NCT02287233), or the HMAs, azacitidine or decitabine (NCT02203773), leading to accelerated FDA approval of these treatment combinations [17-19].

Recent studies conducted with MDM2 inhibitors (MDM2i) in combination with cytarabine have shown safety and activity in AML. In a phase I/Ib study of Idasanutlin (RG7388), a nutlin-class MDM2i, when given in combination with cytarabine, was noted to be safe and showed clinical activity in R/R AML, with 6CRs out of 23 patients enrolled in the dose escalation phase and 4 CRs, 1 CRi, 1 PR, 1HI out of 34 patients enrolled in the dose extension phase [11]. In a recent update of the study, the overall response rate was noted to be 33% with composite CR rate of 29% [12]. Currently a multi-center, double-blind, placebo controlled, phase III study of cytarabine with or without idasanutlin is ongoing (NCT02545283).

In Xenograft models using MOLM-13 AML cell lines, no clear enhancement in activity was noted when DS-3032b was administered concomitantly with cytarabine, compared to DS-3032b alone. Since cytarabine causes cell cycle arrest in S phase and DS-3032b causes cell cycle arrest in G1 phase, one rationale is to use these drugs sequentially, cytarabine followed by DS-3032b. We therefore propose a trial of DS-3032b, in combination with LDACand venetoclax for induction and consolidation treatment of relapsed/refractory AML, followed by DS-3032b monotherapy maintenance in responding patients, in a two-arm phase 2 study. In this trial, cytarabine administration will be followed by DS-3032b. When added, venetoclax will be administered concurrently with LDAC and DS-3032b. Institutional standards will be followed for preparation and handling of cytarabine.

2.2 Overview of investigational treatment, DS3032b – MDM2 inhibitor:

The tumor suppressor p53 protein is a transcription factor that plays a central role in preventing tumor development and progression by inducing cell cycle arrest, apoptosis, or senescence. The gene encoding p53 (*TP53*) suffers disabling somatic mutations or deletions in about 50% of all malignant tumors; *TP53* mutations are less frequent in newly diagnosed leukemia, but are more common in an aberrant karyotype or in patients with relapsed or refractory disease. In tumors expressing wild-type protein, the tumor suppressor function of p53 may be attenuated by other mechanisms, such as over-expression of MDM2, a negative regulator of p53. MDM2 oncoprotein binds to p53 with high affinity and negatively modulates the transcriptional activity and stability of the tumor suppressor. In leukemia with functional p53, inhibition of the MDM2-p53 interaction can restore p53 activity and is expected to offer a novel strategy for therapy. DS-3032b is an orally available and highly selective inhibitor of the MDM2-p53 interaction. It shows antitumor efficacy against tumors with wild-type p53 via activation of the tumor suppressor in a variety of cell culture and xenograft models.

Recently, a phase 1 dose escalation study of DS-3032b as a single agent was conducted in patients with hematological malignancies (NCT02319369) [10]. A total of 38 patients with relapsed/refractory AML or high-risk MDS were enrolled in the study in 5 dose levels: 60mg (7 patients), 90mg (6 patients), 120mg (12 patients), 160 mg (8 patients) and 210 mg (5 patients). Median age was 68.5 years (range 30-88) with approximately 2/3rd patients were >65 years. Maximum tolerated dose (MTD) of DS-3032b was determined to be 160 mg daily in 21/28 day cycle. 93% of the subjects experienced a grade >=3 treatment emergent adverse event (TEAE).
The most common (≥20%) TEAEs of any grade regardless of attribution were nausea (73%), diarrhea (57%) vomiting (33%), fatigue (37%), anemia (33%), thrombocytopenia (33%), neutropenia (20%) hypotension (30%), hypokalemia (23%) and hypomagnesemia (20%). A total of 5 subjects experienced dose limiting toxicities; two subjects in the 160 mg cohort due to grade 3 hypokalemia and grade 3 diarrhea, and three subjects in the 210 mg cohort due to grade 3 nausea and vomiting, grade 2 creatinine elevation/ renal insufficiency, and grade 3 anorexia and fatigue. Preliminary PK results showed plasma exposure (Cmax and AUClast) increased with dose; and approximately 2-fold drug accumulation was observed on Day 15 following the daily oral dosing. Increase in the serum levels of macrophage inhibitory cytokine (MIC-1) as a p53 target gene was used as a circulating pharmacodynamic biomarker, where magnitude of MIC-1 serum level increase corresponded with DS3032 plasma exposure.

Clinical activity of single agent DS-3032b was observed from the reduction in bone marrow blasts by the end of cycle 1 (4 weeks) in 15 of 38 patients. Complete remission was observed in 2 subjects with AML; 1 subject each at 120 mg and at 160 mg, with a remission duration of >4 and >10 months, respectively. One subject with MDS achieved marrow CR with platelet improvement, of 4 months duration, at the 120 mg dose level. Of note, each of these three subjects developed a *TP53* mutation while on treatment, identifying a key resistance pathway with MDM2 inhibition as a single agent. Preliminary efficacy and safety results of DS-3032b monotherapy in AML is favorable and supports further evaluation of DS-3032b in combination approaches.

,

DS-3032B (Milademetan) is a substrate for CYP3A and P-gp. A phase I study in healthy subjects was conducted to evaluate the effect of co-administration of the strong CYP3A4 inhibitors (itraconazole and

posaconazole) on milademetan PK (Study U107). Co-administration of milademetan 100 mg with itraconazole 200 mg at steady state increased milademetan mean Cmax and AUCinf by 8% and 115%. Similarly, posaconazole 200 mg at steady state increased milademetan mean Cmax by approximately 19% and AUCinf by approximately 149%. Therefore, dose of milademetan is recommended to be reduced to half when it is concomitantly administered with strong CYP3A4 inhibitors. *The regularly scheduled dose of milademetan can be resumed after a 3-day washout period following discontinuation of the strong CYP3A inhibitor.*

3. STUDY ELIGIBILITY

3.1 Inclusion Criteria

1) Diagnosis of AML by WHO 2016 criteria. Patients will be divided into 2 arms during the Phase 2 portion:
Arm A: Subjects must have newly diagnosed AML, who are otherwise ineligible for intensive chemotherapy.

Arm B: Subjects must have refractory or relapsed AML

2) TP53 wild-type status on molecular testing performed within the last 3 months.

3) Patient should be >/= 18 years old.

4) Eastern Cooperative Oncology Group (ECOG) performance status ≤ 3.

5) Adequate renal function, defined as: creatinine clearance >/= 30 mL/min, as calculated using the modified Cockcroft-Gault equation, ([{140 - age in years} × {actual weight in kg}] divided by [{72 × serum creatinine in mg/dL} multiply by 0.85 if female]), OR creatinine </= 1.5 x ULN., unless related to leukemic involvement.

6) Adequate hepatic function, defined as: AST/ALT≤3 × ULN; Bilirubin ≤ 1.5 × ULN, unless resulting from hemolysis, Gilbert's disease or considered to be due to leukemic involvement.

7) No gastrointestinal issues to interfere with oral medication absorption.

8) No active uncontrolled infection or comorbidity that would interfere with therapy or place patient at increased risk.

9) Subject (male and female) of childbearing/ reproductive potential must agree to use double-barrier contraceptive measures or avoid intercourse during the study and for 90 days after the last dose of study drug.

10) Subject must sign and date an Institutional Review Board-approved informed consent form (including Health Insurance Portability and Accountability Act authorization, if applicable) before performance of any study-specific procedures or tests.

11) Able and willing to provide bone marrow biopsies/aspirates as requested by the protocol.

12) Willing to undergo malignancy genotyping for *TP53* mutation, insertion, or deletion at screening.

13. Use of hydroxyurea is allowed prior to and during the first cycle of study treatment. 1-2 doses of cytarabine are also permitted if needed for cytoreduction prior to initiating study treatment.

3.2 Exclusion:

1) Patient with t(15;17) karyotypic abnormality or a diagnosis of acute promyelocytic leukemia.

2) Patients who are suitable for and willing to receive intensive chemotherapy.

3) Patient with other malignancy that contains a non-synonymous mutation, insertion, or deletion in the *TP53* gene determined previously or at screening.

4) Prior treatment with MDM2 inhibitor.

5) Presence of central nervous system involvement of leukemia. History of prior leptomeningeal leukemia / disease that has fully resolved is eligible.

6) A second concurrent primary malignancy that has required systemic anti-neoplastic treatment within the previous 6 months, except for localized cancers that have apparently been cured, for example non-melanoma skin cancer, superficial bladder cancer, or carcinoma in situ of the cervix or breast.

7) Any condition that would preclude adequate absorption of DS-3032b, including refractory vomiting, malabsorption, biliary shunt, significant bowel resection, and/or graft-versus-host disease (GVHD) affecting the gut.

8) Any active uncontrolled infection, known human immunodeficiency virus infection, or active hepatitis B or C infection.

9) Any concomitant medical condition that would in the opinion of the Investigator increase the risk of toxicity.

10) Unresolved toxicities from previous anticancer therapy, defined as toxicities (other than alopecia) not yet resolved to NCI-CTCAE v5, Grade </=1 or baseline. Subjects with chronic Grade 2 toxicities may be eligible per discretion of the Investigator and Sponsor (eg, Grade 2 chemotherapy-induced neuropathy).

11) Patient having received Hematopoietic Stem Cell Transplantation (HSCT) within 60 days of the first dose of DS-3032b, is on immunosuppressive therapy post-HSCT at the time of screening, or has clinically significant GVHD (use of topical steroids for ongoing skin GVHD will be permitted).

12) Prolongation of corrected QT interval using Fridericia's method (QTcF) at rest, where the mean QTcF interval is > 450 ms for males or > 470 ms for females based on electrocardiograms (ECGs). Patients with RRBB will be eligible after discussion with PI.

13) Pregnant or breastfeeding.

14) Substance abuse or medical, psychological, or social conditions that, in the opinion of the investigator, may interfere with the subject's participation in the clinical study or evaluation of the clinical study results.

Pregnancy related risks:
Pregnant and lactating women will not be eligible; women of childbearing potential should have a negative pregnancy test prior to entering on the study and be willing to practice methods of contraception throughout the study period. Women do not have childbearing potential if they have had a hysterectomy or are postmenopausal without menses for 12 months. In addition, men enrolled on this study should understand the risks to any sexual partner of childbearing potential and should practice an effective method of birth control. Appropriate birth control will be determined by the treating physician.

4. TREATMENT PLAN

4.1 Study design:

- This is a phase I/II single center, open-label study designed to assess the safety and efficacy of DS-3032b, oral MDM2 inhibitor in combination with LDAC, with or without the addition of venetoclax in patients with newly diagnosed and relapsed/refractory AML.

- The Phase I portion will use a 3+3 study design to identify the recommended phase 2 combination doses. 18 patients will enroll in the phase 1 portion of the study.
- 40 patients will be enrolled in Phase II portion; 20 patients in each Arm A and B respectively.

4.2 Dose regimen:

- Cytarabine will be administered SQ twice daily for 10 days of a 28 day cycle (Days 1-10). Cytarabine can be self-administered by the patient as per institutional standard.

- DS-3032b will be administered orally, with at least 8 ounces of water once daily depending on the cohort: D8-14, D8-21, or D5-7 and D15-17 of a 28 day cycle. An alternative drug administration schedule for dose escalation may be considered based on safety and pharmacodynamics response data collected during dose escalation and upon review by the principal investigators and sponsor. If the patient vomits after taking the dose or misses a dose, they should not make up or double dose the next day.

- Venetoclax will be administered orally daily on D1-14 of a 28 day cycle. Each daily dose of venetoclax should be taken together with approximately 240 mL of water and within 30 minutes after the completion of a meal, preferably a low or moderate-fat breakfast. Subjects should not consume grapefruit or grapefruit products, Seville oranges, or Star fruit within the 3-day period prior to the first venetoclax administration and until the last day of venetoclax is completed due to possible CYP3A mediated metabolic interaction.

4.3 Phase 1 – dose escalation/determination of RP2D:

In this phase, the 3+3 algorithm will be applied for dose escalation. Four dose levels of DS-3032b in combination with LDAC, with or without venetoclax will be tested. Based on safety and tolerability data from Phase I dose escalation study of DS-3032b in patients with hematological malignancies, the starting dose of DS-3032b will be 120mg (dose level 0), in combination with cytarabine.

Initially 3 patients will be treated at dose level 0. The following is a description of the dose escalation rule:

If none of the initial 3 patients in the cohort experience a DLT, then a new cohort of 3 patients will be treated at the next dose level (i.e. Dose Level 1), ie cytarabine plus 200 mg DS-3032b plus venetoclax at 600mg orally daily as shown in Table 1., Further escalations of DS-3032b and venetoclax will follow as in Table 1 based on the overall safety and DLT rates. The dose escalations to MTD and the stopping rules are the following:

- If 1 of the 3 initial patients experiences a DLT, then treat 3 additional patients at the same dose (i.e. Dose Level 0). Escalation will only continue if no more than 1 of the 6 patients experiences a DLT.
- If ≥2/3 or ≥2/6 patients experience DLTs in the first treatment cycle, the MTD has been exceeded. No additional patients will be treated at that dose and no further dose escalation

above that dose either will occur. A new cohort of three patients will be enrolled at the next lower dose of DS-3032b(120mg or 90mg depending on dose level at which MTD was exceeded) if at that dose only three patients were treated.

- If dose level -1 is too toxic (i.e. ≥2/3 or ≥2/6 patients experience DLTs in the first cycle), the study will be stopped.
- MTD is defined as the highest dose at which no more than one patient out of 6 patients experience DLTs in the first cycle.

Table 1: Phase 1 dose reduction and escalation for DS-3032b + LDAC + venetoclax

Dose Level	Cytarabine (mg BID), SubQ	Venetoclax (mg/daily), oral	DS-3032b (mg/daily) , oral
+2	20mg BID x 10 days (D1-10)	600mg daily x 14 days (D1-14)	260mg daily x D5-7 and D15-17
+1	20mg BID x 10 days (D1-10)	600mg daily x 14 days (D1-14)	200mg daily x 7 days (D8-14)
0 (starting dose)	20mg BID x 7 days (D1-7)	----	120mg daily x 14 days (D8-21)
-1	20mg BID x 10 days (D1-7)	----	120mg daily x 7 days (D8-14)

Dose reductions beyond those mentioned in this table or different to the doses specified should be discussed with the PI and documentation of the justification recorded in the chart.

4.4 Dose escalation using alternative drug administration schedule:

Based on the safety and pharmacodynamics response data collected during dose escalation using dose schedule/duration (LDAC D1-7 followed by DS-3032b D8-21, venetoclax D1-14), an alternative drug administration schedule for dose escalation may be considered following review by the principal investigators and study supporter. This will be submitted as a separate protocol amendment.

4.5 Phase 2-Dose expansion:

Upon completion of the Phase 1 portion, the dose expansion phase will begin at the recommended phase 2 dose and drug administration schedule with the intention of confirming safety, tolerability and determining the efficacy of DS-3032b in combination with LDAC, with or without addition of venetoclax in patients with newly diagnosed and relapsed/refractory AML. An additional 40 patients will be enrolled in the Phase 2 portion, 20 patients in each Arm A and B respectively.

4.7 Disease assessment:

Bone marrow biopsies/aspirates and blood samples for disease assessment will be performed according to the study schedule at baseline and on Cycle 2 Day 1 while the subject remains on study. If aplasia is observed on Cycle 2 Day 1, study drugs will be withheld and a confirmation bone marrow assessment will be performed in 2 weeks. For subjects who achieve a complete remission (CR), a follow-up bone marrow evaluation is only required as clinically indicated. All subjects with less than a CR must have a

monthly bone marrow evaluation unless blasts are present in the peripheral blood, in which case bone marrow sampling is only required as clinically indicated (See Table of Events).

4.8 Study duration:

Ongoing study treatment will continue until discontinuation due to relapse, unacceptable toxicity, lack of response, or progressive disease. This is no finite number of cycles received; patients may continue on study so long as they derive clinical benefit from study treatment. This includes:

1. clinically significant progressive disease at any time, or
2. a lack of clinical benefit after 2 induction cycles of treatment, or
3. possibility of undergoing allogeneic stem cell transplantation, or
4. intercurrent illness that prevents further administration of treatment, or
5. unacceptable adverse event(s), or
6. patient decision for study withdrawal, or
7. general or specific changes in the patient's condition that render the patient unacceptable for further treatment in the judgment of the investigator

In addition, if *TP53* genotyping performed during screening shows that a subject's malignancy contains a non-synonymous mutation, insertion, or deletion in the *TP53* gene after DS-3032b administration has begun, the subject will discontinue from the study unless a clinical response is noted upon completion of Cycle 1.

4.9 Dosing delays/dose modifications:

4.9.1 **Toxicity Directly Attributable to Study Drug**

Patients experiencing unacceptable toxicity directly attributable to the study drugs should temporarily stop treatment according to the guidelines in the dose adjustment schema.

4.9.2 Toxicity Grading

Toxicity grading will be according to the NCI CTCAE, v5. To prevent unnecessary morbidity, the following guidelines for dose adjustment for drug-related toxicities are recommended.

4.9.3 Dose reductions

Table 2: Dose adjustments of Cytarabine, DS-3032b and venetoclax for hematological drug-related adverse events

Dose Level	Cytarabine (mg BID), SubQ	Venetoclax (mg/daily), oral	DS-3032b (mg/daily), oral
+2	20mg BID x 10 days (D1-10)	600mg daily x 14 days (D1-14)	260mg/daily D5-7 and D15-17
+1	20mg BID x 10 days (D1-10)	600mg daily x 14 days (D1-14)	200mg/daily x 7 days (D8-14)

0 (starting dose)	20mg BID x 7 days (D1-7)	--	120mg/daily x 14 days (D8-21)
-1	20mg BID x 7 days (D1-7)	--	120mg/daily x 7 days (D8-14)

Dose reductions beyond those mentioned in this table or different to the doses specified should be discussed with the PI and documentation of the justification recorded in the chart.

4.9.4 Dose modifications and interruptions are permitted as necessary for the clinical benefit of the patient. Dose modification and/or discontinuation of either LDAC, DS-3032b and/or venetoclax for patients receiving combination therapy are allowed. If side effects or toxicity are related to one study drug only; patients may discontinue that agent and continue treatment with the alternate study drug (i.e. DS-3032b or venetoclax as monotherapy), if in the investigator's assessment the patient continues to demonstrate clinical benefit and all protocol-specified criteria for continuation of study treatment are met. All dose modifications should be discussed with the PI and clearly documented in the medical record. Treatment may be held as long as clinically necessary for resolution, after discussion with the PI.

4.9.5 Myelosuppression:

Patients with leukemias usually present with abnormal peripheral blood counts at the time therapy is started and myelosuppression is an expected event during the course of therapy for acute leukemias. Thus, no dose adjustments or treatment interruptions for myelosuppression will be planned for the first 4 weeks of therapy. After this time, treatment interruptions and dose adjustments may be considered on an individual basis. The following guidelines can be used to consider treatment:

4.9.5.1 Patients with neutropenia or thrombocytopenia as a consequence of the disease do not require treatment interruptions for myelosuppression. Dose reductions in these patients should be considered in an individual case discussed with the PI. The following guidelines can be used for these patients:

4.9.5.1.1 Patients with a response and pre-cycle counts of neutrophils >1 x10^9 /L and platelets >50 x10^9 /L who have sustained low counts of neutrophils <0.5 x10^9/L or a platelet count <20 x10^9/L for more than 2 consecutive weeks in the current cycle, may receive a subsequent course at 1 dose level reduction. A reduction of 2 dose levels may be considered if the myelosuppression was deemed severe and life threatening by the treating physician, and if it is in the patient's best interest.

4.9.5.1.2 If there are persistent peripheral blood blasts, or the bone marrow shows >5% blasts, may continue treatment regardless of neutrophil and platelet count and give supportive care as needed.

4.9.5.1.3 If no marrow evidence of leukemia, may hold therapy until recovery of granulocytes to ≥1 x10^9 /L and platelets ≥ 60 x10^9 /L, then resume at same or 1 lower dose level according to guidelines mentioned above.

4.9.5.1.4 Based on clinical observations with BCL-2 inhibition, patients treated with venetoclax may experience neutropenia.

If a patient achieves a clinical response including CR, CRi, or MLFS while on study and they have not recovered ANC $\geq$ 500/uL within 14 days of venetoclax drug interruption, unless it is thought to be due to the underlying disease, venetoclax dosing may be further interrupted until ANC recovery to $\geq$ 500/uL. Venetoclax may be reinitiated at a lower dose or reduced duration per cycle (7 days) per discussion with the PI. GCSF may be administered if in the best interest of the patient.

4.9.5 Dose reductions for non-hematologic toxicity possibly related to study drugs should be performed according to the following table:

Toxicity	Grade	Actions
Non-hematological (excludes myalgia/ arthralgia responding to treatment, inadequately treated nausea, vomiting and diarrhea, or electrolyte abnormalities unless not responding to optimal supplementation)	>=3	Hold therapy until recovery to Grade ≤ 1, then re-start and reduce to the next lower dose level. If toxicity recurs again, hold therapy until recovery to grade ≤1, then re-start and reduce one additional dose level. Dose reductions below dose level -1 will be considered on an individual basis after discussion with the principal investigator.
Non-hematological, study-drug related	4	Remove from study. An exception can be made if grade 4 toxicity reverses to Grade ≤ 1 on or before 14 days of interrupting study drug and re-dosing with MDM2i is considered safe and in the best interest of the patient. In those instances treatment may be resumed with a 1 dose-level reduction after discussion with PI and Sponsor.
Non-hematological (excludes myalgia/ arthralgia responding to treatment, inadequately treated vomiting and diarrhea, or electrolyte abnormalities unless not responding to optimal supplementation)	Persistent 2 considered clinically significant or upon patient's request	Hold therapy until recovery to Grade ≤ 1, then re-start and reduce to the next lower dose level. If toxicity recurs again, hold therapy until recovery to grade ≤1, then re-start and reduce one additional dose level. Dose reductions below dose level -1 will be considered on an individual basis after discussion with the principal investigator.

4.9.5.1 Patients in whom the toxicity occurs or persists beyond the planned completion of drug administration for the cycle will have the dose reductions implemented in subsequent cycles provided the toxicity has resolved as specified in the table above.

4.9.5.2 If adverse events are clearly associated with one of the drugs, dose adjustments to only that agent may be performed.

4.9.5.3 Dose modifications different than those described above (e.g., dose reductions by 2 dose levels, decreased number of days of administration of either drug, continuation of only DS-3032b) may be implemented if judged in the best interest of the patient after discussion with the PI and documentation of the rationale for this action.

4.9.5.4 Dose adjustments for only one of the drugs may be considered when the adverse event may be clearly associated with only one drug.

5. STUDY PROCEDURES (see Table of Events)

5.1 Screening:

5.1.1 Screening (Pre-cycle)

The screening (pre-cycle) period occurs within 14 days prior to starting study therapy.

The following procedures will be performed during the screening period:

- Obtain written (ie, signed and dated) informed consent.
- Perform a complete physical examination and record weight and height.
- Obtain vital sign measurements (systolic and diastolic blood pressure, pulse rate, and body temperature).
- Assess functional status using the ECOG Performance Status Scale.
- Obtain blood samples including CBC/diff, chemistry (including LFTs), uric acid and coagulation profile (PTT; INR/PT)
- Obtain a blood sample for biomarkers.
- Obtain a serum or urine sample for pregnancy testing in women of childbearing potential.
    - Obtain urine analysis.
- Perform a 12-lead ECG.
- Record concomitant medications.
    - Concomitant medication will be captured in subjects medical record only
- Assess subjects for adverse events.

• Obtain bone marrow biopsies/aspirates for marrow assessment and biomarker test. A bone marrow evaluation is required for all subjects at screening.

5.2 Treatment Period

5.2.1 Cycle 1/Day -1 (for dose level +1 and beyond)

The patient will be admitted to the hospital for the venetoclax ramp-up period during Cycle 1. Hospitalization will begin by study day -1, until at least 24 hours after reaching the designated dose of venetoclax. To mitigate the risk for TLS, subjects must be receiving tumor lysis prophylaxis, including hydration (oral, intravenous) and treatment with a uric acid reducing agent (allopurinol, rasburicase) per institutional guidelines prior to and during the venetoclax ramp-up period of Cycle 1.

5.2.2 Cycle 1/Day 1(+/- 2 days)

• Perform a complete physical examination and record weight.

• Obtain vital sign measurements (systolic and diastolic blood pressure, pulse rate, and body temperature).

• Obtain blood samples including CBC/diff,chemistry and uric acid.

• Obtain a blood sample for biomarkers.

• Record concomitant medications.

• Assess subjects for adverse events.

• Administer LDAC twice daily Day 1-7/28 (dose level 0); Day 1-10 (dose level +1 and beyond) per protocol.

• Administer venetoclax daily Day 1-14/28 per protocol **(for dose level +1 and beyond)**

Venetoclax Ramp-Up:

At dose level +1 and +2, venetoclax will be administered at a dose of 100 mg on day 1, 200 mg on day 2, and 400 mg on day 3, and 600mg on day 4-14 of the first cycle.

During cycle 1 venetoclax dosing ramp up, TLS lab monitoring (K+, uric acid, Phosphorus, Calcium and Creatinine) should occur at pre- dose and 24 hours post dose (inpatient AM labs may substitute for the 24 hour post-dose labs). Patients will be admitted to the hospital for the venetoclax ramp-up period of cycle 1.

5.2.3 Cycle 1/Day 8 (dose level 0 and +1) (+/- 2 days)

The following procedures will be completed during Cycle 1/Day 8 visit.

• Perform a complete physical examination and record weight.

• Obtain vital sign measurements (systolic and diastolic blood pressure, pulse rate, and body temperature).

• Obtain blood samples including CBC/diff,chemistry and uric acid.

• Obtain a blood sample for biomarkers.

• Record concomitant medications.

• Assess subjects for adverse events.

• Administer LDAC twice daily Day 1-10 (dose level +1 and beyond) per protocol.

• Administer venetoclax daily Day 1-14/28 per protocol.

• Administer DS-3032b for QD Day 8-21/28 (dose level 0); day 8-14/28 (dose level +1) per protocol.

- For dose level +2, there will be a cycle1/Day 5 visit and DS3032b will be administered Day 5-7 and Day 15-17/28 days per protocol. There will not be a cycle 1/Day 8 visit for dose level +2.

5.2.4 Cycle 1/Day 15 (+/- 2 days)

The following procedures will be performed during Cycle 1/Day 15 visit

• Perform a complete physical examination and record weight.

• Obtain vital sign measurements (systolic and diastolic blood pressure, pulse rate, and body temperature).

• Obtain blood samples including CBC/diff,chemistry and uric acid.

• Record concomitant medications.

• Assess subjects for adverse events. • Administer DS-3032b for QD Day 8-21/28 (dose level 0); Day 15-17 (dose level +2) per protocol.

5.2.5 Cycle 1/Day 22 (+/- 2 days)

The following procedures will be completed during Cycle 1/Day 22 visit:

• Perform a complete physical examination and record weight.

• Obtain vital sign measurements (systolic and diastolic blood pressure, pulse rate, and body temperature).

• Obtain blood samples including CBC/diff,chemistry and uric acid.

• Record concomitant medications.

• Assess subjects for adverse events.

5.2.6 Cycle 2/Day 1 (± 3 days)

• Perform a complete physical examination and record weight.

• Obtain vital sign measurements (systolic and diastolic blood pressure, pulse rate, and body temperature).

• Obtain blood samples including CBC/diff and chemistry

• Obtain a blood sample for biomarkers.

• Record concomitant medications.

• Assess subjects for adverse events.

• Obtain bone marrow biopsies/aspirates for marrow assessment and/or biomarker tests.

• Administer LDAC twice daily Day 1-7/28 per protocol.

5.2.7 Cycle 3 and all subsequent cycles, Day 1 (± 7 days)

The following procedures will be performed at Cycle 3/Day 1 visit

• Perform a complete physical examination and record weight.

• Obtain vital sign measurements (systolic and diastolic blood pressure, pulse rate, respiratory rate, and body temperature).

• Obtain blood samples including CBC/diff and chemistry (including LFTs)

• Obtain a blood sample for biomarkers.

• Record concomitant medications.

• Assess subjects for adverse events.

• Obtain bone marrow biopsies/aspirates for marrow assessment and/or biomarker tests after C3 (C4D1) and then after every odd cycle till CR. Once in CR, additional bone marrow exams per clinical discretion. Bone marrow biomarkers will be obtained with each bone marrow performed.

5.2.8 End-of-Treatment (Post-cycle)

This End-of-Treatment Visit should occur at the earliest day possible within 30 days after the last administration of DS-3032b, but before beginning any other form of anticancer therapy. The following assessments will be performed at this visit:

• Perform a complete physical examination and record weight and height.

• Obtain vital sign measurements (systolic and diastolic blood pressure, pulse rate, and body temperature).

• Obtain blood samples including CBC/diff and chemistry

• Obtain a serum or urine sample for pregnancy testing in women of childbearing potential.

• Obtain a blood sample for biomarkers.

• Record concomitant medications.

• Assess subjects for adverse events.

• Record reason for treatment discontinuation.

• Bone marrow re-biopsy for subjects who achieved an initial CR/PR to DS-3032b but later developed progressive disease while on therapy.

5.3 Identification of Investigational Product:

International Non-proprietary name: DS-3032b

Manufacturer: Daiichi Sankyo Pharma Development

Dose: 120 – 260 mg

Route of Administration: oral

Formulation: 30mg and 100mg DS-3032b capsules which are individually packaged in desiccant-embedded aluminum blisters

DS-3032b will be provided by Daiichi Sankyo Pharma Development. All expired/unused study drug will be destroyed per the institution policy.

LDAC and venetoclax will be obtained commercially.

5.4 Supportive care:

Supportive measures including blood and platelet transfusions, prophylactic and therapeutic antimicrobials, and analgesics are permitted. Growth factor use is permitted on study at the discretion of the treating physician if in the best interest of the patient. Electrolyte abnormalities will be addressed prior to starting therapy.

Tumor Lysis Prophylaxis:

The venetoclax dose titration scheme utilized in AML studies to date to mitigate the risk of tumor lysis syndrome (TLS) will be employed. All patients will be hospitalized for the entirety of the venetoclax dose escalation ramp up starting at least on day -1 of treatment initiation and until 24 hours after the completion of venetoclax dose escalation.

To mitigate the risk for TLS, subjects must be receiving tumor lysis prophylaxis, including hydration (oral, intravenous) and treatment with a uric acid reducing agent (allopurinol, rasburicase) as per institutional standards prior to and during the venetoclax ramp up period of Cycle 1. Patients with reduced renal clearance and/or splenomegaly are at increased risk to develop TLS.

TLS chemistry tests (potassium, uric acid, creatinine, calcium and phosphorus) will be obtained prior to dosing and 24 hours after dosing each day of a new venetoclax dose. TLS chemistry test results will be reviewed by the investigator and prior to the subject's next venetoclax dose to ensure appropriate management. If a subject meets criteria for clinically significant laboratory or clinical TLS, no additional venetoclax should be administered until resolution.

Venetoclax interruption for up to 72 hours following transient (< 48 hours) chemical changes and laboratory TLS will be allowed and will not require a dose reduction and is not considered a DLT.

CYP3 Inducers and Inhibitors:

Moderate and strong CYP3A inhibitors including isavuconazole and posaconazole are allowed. If a moderate CYP3A inhibitor is prescribed on study, a 50% dose reduction of VEN will be required (i.e. 300 mg instead of 600 mg, etc). If a strong CYP3A inhibitor is prescribed on study, a 75% dose reduction of VEN will be required (i.e. 150 mg instead of 600 mg, etc). With concomitant posaconazole use, the dose of VEN should be reduced to 70mg. For DS3032b, no dose reduction is required for moderate CYP3A inhibitors. For strong CYP3A inhibitors, a 50% dose reduction of DS3032b will be required (i.e. 60mg instead of 120mg, etc). *The regularly scheduled dose of DS-3032b (milademataan) can be resumed after a 3-day washout period following discontinuation of the strong CYP3A inhibitor.*

Table of Events

Cycle	Pre-cycle	1(+/- 2 days)					2 (± 3 days)		3 and beyond (±7 days)	End of study (within 30 days of last dose of DS-3032b)
Cycle Day (s)	-14 to 0	-1 (dose level +1 and above)	1	8[d]	15	22	1		1	
Informed consent	x									
Pregnancy test	x									
Adverse events	x		x	x	x	x	x		x	x
Prior/concomitant medications	x		x	x	x	x	x		x	x
Tumor lysis syndrome prophylaxis[a]		x	x	x	x	x				
ECOG	x									
Physical examination, including vitals, height and weight	x		x	x	x	x	x		x	x
CBC/diff[b]	x		x	x	x	x	x		x	x
Chemistries and uric acid[b]	x		x	x	x	x	x		x	x
Coagulation panel	x									
Urinalysis	x									
EKG	x									
Peripheral Blood Biomarkers	x		x	x			x		x	x
Bone marrow assessment [c]	x						x		x	x
End of study bone marrow assessment										x

a. All subjects must receive tumor lysis prophylaxis prior to and during treatment. For details on tumor lysis prophylaxis and management, refer to Section on Management of Tumor Lysis Syndrome and index A.

b. CBC differential may be omitted if WBC $\leq$ 0.5 x 10^9/L. During cycle 1 venetoclax dosing ramp up, TLS lab monitoring (K+, uric acid, Phosphorus, Calcium and Creatinine) should occur at pre- dose and 24 hours post dose (inpatient AM labs may substitute for the 24 hour post-dose labs).

c. Obtain bone marrow biopsies/aspirates for marrow assessment and/or biomarker tests after C1 (C2D1), after C3 (C4D1) and then after every odd cycle till achieving CR. Once in CR, additional bone marrow exams per clinical discretion. Bone marrow biomarkers will be obtained with each bone marrow performed.

d. At dose level +2, there will be a Cycle 1 Day 5 visit instead of a Day 8 visit.

6. RESPONSE DEFINITIONS

6.1 Study endpoints:

6.1.2 Primary Efficacy Analysis:

Clinical activity of LDAC in combination with DS-3032b, with or without the addition of venetoclax will be assessed based on the 2017 ELN recommendations for AML, with an overall response rate (ORR) defined as CR + CRi +PR+MLFS.

17.5. Response Criteria for AML

Category	Definition
Complete Remission (CR)	Bone marrow blasts < 5%; absence of circulating blasts and blasts with Auer rods; absence of extra-medullary disease; ANC $\geq 1.0 \times 10^9/L$; platelet count $\geq 100 \times 10^9/L$
CR with Incomplete Blood Count Recovery (CRi)	All CR criteria except for ANC < $1.0 \times 10^9/L$ OR platelet count < $100 \times 10^9/L$
CR with Partial Hematological Recovery (CRh)*	All CR criteria except for ANC > $0.5 \times 10^9/L$ AND platelet count >$50 \times 10^9/L$
Partial Remission (PR)	Decrease of bone marrow blast percentage by at least 50% to a value of 5% to 25% and ANC $\geq 1.0 \times 10^9/L$; platelet count $\geq 100 \times 10^9/L$
Morphologic Leukemia-Free State (MLFS)	Bone marrow blasts < 5%; absence of blasts with Auer rods; absence of extra-medullary disease; no hematologic recovery required
Stable Disease (SD)	Absence of CR, CRi, CRh, PR, or MLFS for at least 3 months; and criteria for PD not met
Progressive Disease (PD)	Evidence for an increase in bone marrow blast percentage and/or increase of absolute blast counts in the blood:<br>• 50% increase in marrow blasts over baseline (a minimum 15% point increase is required in cases with < 30% blasts at baseline; or persistent marrow blast percentage of > 70% over at least 3 months; without at least a 100% improvement in ANC to an absolute level of > $0.5 \times 10^9/L$, and/or platelet count to > $50 \times 10^9/L$ (non-transfused); or<br>• 50% increase in peripheral blasts (WBC x % blasts) to > $25 \times 10^9/L$ (in the absence of differentiation syndrome); or<br>• New extra-medullary disease

ANC = absolute neutrophil count; WBC = white blood cells.
Response criteria modified based on Cheson et al 2003 and Dohner et al 2017.[15,16]
* If a subject meets the criteria for both CRi and CRh, the outcome should be captured in the CRF as CRh.

Blood cell count criteria for CRi (without overlap with CRh) will be ANC $\geq 1.0 \times 10^9/L$ and platelet count $\leq 50 \times 10^9/L$, or ANC $\leq 0.5 \times 10^9/L$ and platelet count $\geq 100 \times 10^9/L$.

Additional analyses of response including CR/CRi rate and MLFS rate will be performed for enrolled subjects. The depth of remission such as with exploratory analyses of MRD negativity by flow cytometry and/or molecular analysis will also be performed.

For each subject, response to therapy, duration of response, event-free survival, and overall survival will be calculated. The duration of response is defined as the number of days from the date of initial

response (PR or better) to the date of first documented disease progression/relapse or death, whichever occurs first. Event-free survival is defined as the number of days from the date of treatment initiation (i.e., C1D1) to the date of documented treatment failure, relapses from CR, or death from any cause, whichever occurs first, and will be calculated for all patients. In the event that neither disease progression nor death is documented prior to study termination, analysis cutoff, or the start of confounding anticancer therapy, these endpoints will be censored at the date of last tumor assessment date.

In addition, relationships between anti-leukemia activity / efficacy, pharmacodynamic markers, exploratory biomarkers, and drug exposure levels will be explored.

6.1.3 Primary Safety Endpoint:

The primary safety endpoint will include serious adverse events (SAEs), TEAEs, DLTs, physical examination findings (including ECOG performance status), vital sign measurements, clinical laboratory parameters (serum chemistry, hematology, and urinalysis), and electrocardiogram (ECG) parameters, particularly the QTcF. Adverse events will be categorized using the Medical Dictionary for Regulatory Activities (MedDRA). Adverse events and laboratory test results will be graded using the NCI-CTCAE version 5.0.

An adverse event is the appearance or worsening of any undesirable sign, symptom, or medical condition occurring after starting the study drug even if the event is not considered to be related to study drug. Medical conditions/diseases present before starting study drug are only considered adverse events if they worsen after starting study drug. Abnormal laboratory values or test results, even when they do not induce clinical signs or symptoms, or require therapy, are considered clinically significant. .Adverse events (AEs) will be collected using the Leukemia-Specific Adverse Event Recording and Reporting Guidelines.

Adverse drug reaction is a response to a drug which is noxious and unintended and which occurs at doses normally used in man for prophylaxis, diagnosis, or therapy of disease or for the modification of physiologic function.

All “suspected adverse reactions” (as defined in 21 CFR 312.32(a)) will be captured in the case report forms (CORe and PDMS). For abnormal chemical values grade 3 or 4, the apogee will be reported per course in the CRF.

Assessing causal connections between agents and disease is fundamental to the understanding of adverse drug reactions. In general, a drug may be considered a contributory cause of an adverse event if, had the drug not been administered, 1) the event would not have happened at all, 2) the event would have occurred later than it actually did, or 3) the event would have been less severe.

The Investigator or physician designee is responsible for verifying and providing source documentation for all adverse events and assigning the attribution for each event for all subjects enrolled on the trial.

6.1.4 Monitoring, recording and reporting adverse events

All subjects will be monitored for AEs during the study. Assessments may include monitoring of any or all of the following parameters: the subject's clinical symptoms, laboratory, pathological, radiological or surgical findings, physical examination findings, or findings from other tests and/or procedures.

AEs will be recorded in the subject's source documents by the Investigator from the first dose through 30 days after the last dose. Serious Adverse Events (SAEs) will be captured starting from the first protocol intervention. The Investigator or physician designee is responsible for verifying and providing source documentation for all adverse events and assigning the attribution for each event for all subjects enrolled on the trial.

The Principal Investigator will sign and date the adverse event logs per patient at the completion of each course. Following signature, the adverse event logs will be used as source documentation for the adverse events for attribution.

The Leukemia-specific Adverse Event Recording and Reporting Guidelines will be followed for the recording and reporting of adverse and serious adverse events.

1. Baseline events will be recorded in the medical history section of the case report form and will include the terminology event name, grade, and start date of the event. The medical history section of the case report form will serve as the source document for baseline events once signed and dated by the principal investigator.
   Baseline events are any medical condition, symptom, or clinically significant lab abnormality present before the informed consent is signed
   i. Hematologic laboratory abnormalities will not be recorded as baseline events for patients with acute leukemia, myelodysplastic syndrome, chronic lymphocytic leukemia, or chronic myeloid leukemia in blast phase.
   ii. If exact start date is unknown, month and year or year may be used as the start date of the baseline event.
2. The maximum grade of the adverse event will be captured per course or protocol defined visit date.
3. These adverse events will be recorded in the case report form:
   a. Any grade adverse event that is possibly, probably, or definitely related to the study drug(s).
   b. All serious adverse events regardless of attribution to the study drug(s).
   c. Any grade adverse event regardless of attribution to the study drug(s) that results in any dose modification.
4. Hematologic adverse events will not be recorded or reported for studies in patients with acute leukemia, myelodysplastic syndrome, chronic lymphocytic leukemia, or chronic myeloid leukemia in blast phase except for:
   a. Prolonged myelosuppression as defined by the NCI-CTCAE criteria specific for leukemia, e.g. marrow hypocellularity on day 42 or later (6 weeks) from start of therapy without evidence of leukemia (< 5% blasts), or that results in dose modifications, interruptions or meets the protocol definition of DLT or SAE.
5. Serious adverse events will be reported according to institutional policy.
6. Protocol specific language regarding the recording and reporting of adverse and serious adverse events will be followed in the event of discordance between the protocol and Leukemia-specific

adverse event recording and reporting guidelines.

The descriptions and grading scales found in the revised NCI Common Terminology *Criteria for Adverse Events (CTCAE) version 5.0* will be utilized for adverse event reporting. *(http://ctep.cancer.gov/reporting/ctc.html).*

6.1.5 Definition of Dose-Limiting Toxicity

A dose-limiting toxicity (DLT) is defined as any clinically significant non-hematologic treatment-emergent adverse event (TEAE) or abnormal laboratory value that is Grade 3 or higher according to National Cancer Institute (NCI) Common Terminology Criteria for Adverse Events (CTCAE) v5.0 occurring during the first cycle on study that cannot be attributed by the investigator to a clearly identifiable cause such as disease progression, underlying illness, concurrent illness, or concomitant medication, with the following exceptions:

For elevations in hepatic function enzymes, a DLT is defined as follows:

1. Grade 4 aspartate aminotransferase (AST)/alanine aminotransferase (ALT) levels
2. AST/ALT $> 5 \times$ the upper limit of normal (ULN) lasting > 3 days
3. AST/ALT $> 5 \times$ ULN if accompanied by $\geq$ Grade 2 elevation in bilirubin

The following events are classified as DLTs:

1. Subjects who are unable to complete at least 75% of the prescribed dose (ie, 10 days) of DS-3032b in the first cycle as a result of non-disease related Grade ≥ 2 events will be considered to have a DLT.
2. Failure to recover a peripheral absolute neutrophil count $\geq 500/mm^3$ and platelets $\geq 20 \times 10^9$ /L in the setting of a hypocellular bone marrow without evidence of residual leukemia, resulting in a > 2-week delay in initiating Cycle 2.
3. Any grade 3 nausea, vomiting or diarrhea that requires TPN or tube feeding.

The following adverse events are NOT considered DLTs:

1. Grade 3 fatigue lasting < 3 days
2. Grade 3 nausea or vomiting that has resolved to ≤Grade 2 within 48 hours following administration of preventive, as well as additional therapeutic antiemetic agents for managing established nausea or vomiting (eg droperidol, aprepitant, etc.)Grade 3 diarrhea that has resolved to Grade ≤ 2 within 48 hours after standard antidiarrheal therapies
3. Isolated laboratory findings not associated with signs or symptoms including Grade 3 or4 alkaline phosphatase, uric acid, amylase, and lipase elevations, and Grade 3 hyponatremia lasting < 72 hours developed from Grade 1 at baseline.
   Venetoclax interruption for up to 72 hours following transient (< 48 hours) chemical changes and laboratory TLS may be allowed and may not be considered a DLT.

4. Grade ≥3 electrolyte abnormalities should only be considered as exceptions to the DLT criteria if they are both correctable within a fixed time period of no more than 72 hours and are clinically asymptomatic.

Hematologic DLT is defined as Grade $\geq$ 3 neutropenia and/or thrombocytopenia, with a hypocellular bone marrow and no greater than 5% marrow blasts lasting for 6 weeks or more after the start of a course in the absence of underlying MDS. Anemia will not be considered for the definition of DLT.

Patients with neutropenia or thrombocytopenia as a consequence of the disease prior to the start of therapy do not require treatment interruptions for myelosuppression. Dose reductions of the study treatment in these patients should be considered on an individual case basis and discussed with the PI.

6.1.6 Definition of Serious Adverse Events (SAEs)

An adverse event or suspected adverse reaction is considered "serious" if, in the view of either the investigator or the sponsor, it results in any of the following outcomes:

- Death
- A life-threatening adverse drug experience – any adverse experience that places the patient, in the view of the initial reporter, at immediate risk of death from the adverse experience as it occurred. It does not include an adverse experience that, had it occurred in a more severe form, might have caused death.
- Inpatient hospitalization or prolongation of existing hospitalization
- A persistent or significant incapacity or substantial disruption of the ability to conduct normal life functions.
- A congenital anomaly/birth defect

Important medical events that may not result in death, be life-threatening, or require hospitalization may be considered a serious adverse drug experience when, based upon appropriate medical judgment, they may jeopardize the patient or subject and may require medical or surgical intervention to prevent one of the outcomes listed in this definition. Examples of such medical events include allergic bronchospasm requiring intensive treatment in an emergency room or at home, blood dyscrasias or convulsions that do not result in inpatient hospitalization, or the development of drug dependency or drug abuse (21 CFR 312.32).

- Important medical events as defined above may also be considered serious adverse events. Any important medical event can and should be reported as an SAE if deemed appropriate by the Principal Investigator or the IND Sponsor, IND office.

- All events occurring during the conduct of a protocol and meeting the definition of a SAE must be reported to the IRB in accordance with the timeframes and procedures outlined in "The University of Texas M. D. Anderson Cancer Center Institutional Review Board Policy for Investigators on Reporting Serious Unanticipated Adverse Events for Drugs and Devices". Unless

stated otherwise in the protocol, all SAEs, expected or unexpected, must be reported to the IND Office, regardless of attribution (within 5 working days of knowledge of the event).

- **All life-threatening or fatal events,** that are unexpected, and related to the study drug, must have a written report submitted within **24 hours** (next working day) of knowledge of the event to the Safety Project Manager in the IND Office.
- Unless otherwise noted, the electronic SAE application (eSAE) will be utilized for safety reporting to the IND Office and MDACC IRB.
- Serious adverse events will be captured from the time of the first protocol-specific intervention, until 30 days after the last dose of drug, unless the participant withdraws consent. Serious adverse events must be followed until clinical recovery is complete and laboratory tests have returned to baseline, progression of the event has stabilized, or there has been acceptable resolution of the event.
- Additionally, any serious adverse events that occur after the 30 day time period that are related to the study treatment must be reported to the IND Office. This may include the development of a secondary malignancy.

Reporting to FDA:

- Serious adverse events will be forwarded to FDA by the IND Sponsor (Safety Project Manager IND Office) according to 21 CFR 312.32.

It is the responsibility of the PI and the research team to ensure serious adverse events are reported according to the Code of Federal Regulations, Good Clinical Practices, the protocol guidelines, the sponsor's guidelines, and Institutional Review Board policy.

6.1.7 Biomarker Endpoints (non-optional):

Peripheral blood and bone marrow aspirate samples will be obtained at study specified time points. Unused cells will be frozen and stored.

NGS molecular sequencing in all patients

As a standard of care all AML patients at MDACC are evaluated for karyotype and molecular mutation profile using a CLIA-certified next-generation gene panel sequencing platform. The effects of the combination will be compared not only to historical outcomes in matched groups of patients, but also at the molecular and cellular level by correlating with karyotype and molecular mutation profile. The NGS gene panel will be performed on the screening BM aspirate and the progression/relapse BM aspirate. An aliquot of DNA will be stored for additional analysis of DNA mutations in responding patients.

***TP53* genotyping:**

TP53 genotyping will be performed on whole sample or on enriched leukemic cells from peripheral blood and/or bone marrow samples from all enrolled subjects. Confirmation of *TP53* wild-type status from the screening analysis is NOT required prior to DS-3032b dosing, but the Investigator and subject will be informed about the genotyping result as soon as it is available. If the testing result shows that a

subject's malignancy contains a non-synonymous mutation, insertion, or deletion in the *TP53* gene after DS-3032b administration has begun, the subject will discontinue from the study unless a clinical response is noted upon completion of Cycle 1. If the *TP53* testing result returns as indeterminate, the subject can still continue the study if clinical benefit is noted. *TP53* re-testing can be considered.

Genomic DNA (gDNA) is extracted from fresh bone marrow (BM) aspirate or peripheral blood (PB) using ReliaPrep™ Large Volume HT gDNA Isolation System and quantified using a Qubit dsDNA Broad Range Assay Kit (Thermo Fisher Scientific). Either entire coding sequences or limited exons/hotspots for 81-gene target genes, including TP53 TP53 with the following codons (exons) covered, 2 (1-25), 4-11 (80-394) is captured using the hybridization-based HaloPlex HS target enrichment system by Agilent Technologies. 250 ng gDNA is fragmented using restriction enzymes. A probe library is added to guide the targeted fragments to form circular DNA molecules. The probes are complementary to the 5'- and 3'- ends of a targeted DNA restriction fragment, and contain sequencing-specific motifs. The circular DNA molecules are annealed and amplified by polymerase chain reaction (PCR). PCR amplification generates linear barcoded fragments, which are subject to sequencing. Bidirectional paired-end sequencing is performed using the Illumina MiSeq platform. A minimum of 250x coverage depth is required for any target region for the interpretation.
TP53 gene coding sequences (exons) are captured using the hybridization-based HaloPlex HS target enrichment system (Agilent Technologies). A total of 250 ng gDNA is fragmented using restriction enzymes. A probe library is added to guide the targeted fragments to form circular DNA molecules. The probes are complementary to the 5'- and 3'- ends of a targeted DNA restriction fragment, and contain sequencing-specific motifs. The circular DNA molecules are annealed and amplified by polymerase chain reaction (PCR). PCR amplification generates linear barcoded fragments, which are subject to sequencing. Bidirectional paired-end sequencing is performed using the Illumina MiSeq platform. A minimum of 250x coverage depth is required for any target region for the interpretation. Overall, the assay covers 98% of TP53 coding sequence (exons 2 and, 4 to 11) and >99.5% of all reported TP53 mutations (COSMIC database v86) at the desired coverage of 250x or better.

Analytical validity: A total of 65 patient samples with known TP53 mutation status as tested by an alternative test platform were included in validation. This cohort included 22 samples with TP53 mutations and 43 samples that were wild-type for TP53. The known variants were successfully detected at the level as low as 5% VAF (one mutant allele in the background of nineteen wild type alleles) in all 22 samples. Overall, all 65 samples showed expected TP53 genotype (wild-type or mutant) showing a 100% concordance with the alternative test method (NGS, Sanger sequencing, and/or Pyro-Sequencing) at analytical sensitivity of 5%.

Since the assay is utilized for AML patients with elevated blast count, the combination of elevated blasts and 5% analytical sensitivity will prevent any false-negative results. In addition, based on the clinical experience with this assay which utilizes unique molecular identifiers (UMI) for error correction, mutations in VAF range of 2% to 5% are routinely detected and reported with a comment about low

(<5%) VAF after manual review and confirmation by a MD pathologist to prevent false negative calls in cases with low tumor burden.

The limit of detection (LOD) of this panel was assessed by sequencing 2 cell lines and one patient sample. The cell lines were diluted in a negative control sample (human female DNA, Promega) at 5 different dilutions factors of 100%, 50%, 20%, 10% and 5%. The positive patient sample was diluted into negative patient sample at 6 different dilution factors of 100%, 50%, 20%, 10%, 5% and 2.5%. A total of 151 different single nucleotide variations (SNVs) or indels in 5 different genes at various variant allelic frequencies were known in the cell lines and the patient sample consisting of 65 samples. The known variants were successfully detected at the level of 5% VAF (one mutant allele in the background of nineteen wild type alleles) taking into consideration the depth of coverage at a given base, establishing an analytical sensitivity of 99.3%. Out of the 151 variants, 150 were concordant to the calls made by an alternate chemistry, TruSeq, HaloPlex, Sanger, and Pyro-Sequencing. The variants include 135 single nucleotide polymorphisms (SNPs), and 16 insertion/deletions (INDELS). The missed call was due to low variant allelic frequency, below that assay sensitivity establishing an overall accuracy of 99.3%.

All reagents obtained by the CAP-accredited/CLIA-certified lab are stored and handled as recommended by the manufacturer and records of refrigerator, freezer, and room temperature monitoring is routinely kept. All reagents and chemicals are used within their indicated expiration date. New reagent lots and shipments are checked against old reagent lots using reference material such as a positive control, previously performed patient samples or concurrently prior to placing the reagents in service. For non-critical reagents a concurrent lot validation is performed. The process involves documenting the last run using the old lot and the first run using the new lot. Parallel validation is performed for critical reagents. This involves running two patient samples that have previously been sequenced for the panel within the first run of the new lot. The raw data from the two previously sequenced patient samples performed on both the old and new lot are reviewed by a supervisor and must passed all performance QC metrics as defined by the validation study. Records of the acceptability studies for the new reagents lot and shipments are maintained within the laboratory.

Minimal Residual Disease assessment by flow-cytometry

As a standard of care all BM aspirates will be evaluated for MRD by validated (to a level of at least 0.1%) 17-color multiplanar flow-cytometry.

Gene expression and methylation signatures by RNA sequencing and/or gene expression arrays

This will be performed on PB and/or BM aspirates at screening, end of cycle 1, end of cycle 3, and at time of progression / end of study.

CyTOF analysis (MDACC)

We will analyze MDM2 family expression, stem cell surface markers, differentiation markers, and intracellular signaling markers in AML cells by CyTOF (mass cytometry) assay established in Dr Andreeff laboratory, on PB and/or BM at screening, end of cycle 1, end of cycle 3 and at the time of progression / end of study.

Additional relationships between anti-leukemia activity / efficacy, PDn markers and exploratory biomarkers will be explored.

7. Regulatory and Reporting Requirements

7.1. Informed Consent

Before a subject's participation in the clinical study, the investigator is responsible for obtaining written informed consent from the subject or legally acceptable representative after adequate explanation of the aims, methods, anticipated benefits, and potential hazards of the study and before any protocol-specific screening procedures or any investigational products are administered. A legally acceptable representative is an individual or other body authorized under applicable law to consent, on behalf of a prospective subject, to the subject's participation in the clinical study.

The acquisition of informed consent should be documented in the subject's medical records, and the informed consent form should be signed and personally dated by the subject or a legally acceptable representative and by the person who conducted the informed consent discussion. The original signed informed consent form should be retained in accordance with institutional policy, and a copy of the signed consent form should be provided to the subject or legally acceptable representative.

7.2. Independent Ethics Committee/Institutional Review Board

A copy of the protocol, proposed informed consent form, other written subject information, and any proposed advertising material must be submitted to the IEC/IRB for written approval. A copy of the written approval of the protocol and informed consent form must be received by Daiichi Sankyo before recruitment of subjects into the study and shipment of investigational product.

The investigator must submit and, where necessary, obtain approval from the IEC/IRB for all subsequent protocol amendments and changes to the informed consent form. The investigator should notify the IEC/IRB of deviations from the protocol or serious adverse events occurring at the site and other adverse event reports received from Daiichi Sankyo, in accordance with local procedures.

The investigator will be responsible for obtaining annual IEC/IRB approval/renewal throughout the duration of the study. Copies of the investigator's reports and the IEC/IRB's continuance of approval must be sent to Daiichi Sankyo.

7.3 Subject Confidentiality

The investigator must ensure that the subject's confidentiality is maintained. On documents submitted to Daiichi Sankyo, subjects should be identified by their study number only. Documents that are not for submission to Daiichi Sankyo (eg, signed informed consent forms) should be kept in strict confidence by the investigator.

In compliance with ICH GCP Guidelines, it is required that the investigator and institution permit authorized representatives of the company, of the regulatory agency(s), and the IEC/IRB direct access to review the subject's original medical records for verification of study-related procedures and data. Direct access includes examining, analyzing, verifying, and reproducing any records and reports that are important to the evaluation of the study. The investigator is obligated to inform and obtain the consent of the subject to permit named representatives to have access to his/her study-related records without violating the confidentiality of the subject.

7.4. Study Termination

Both Daiichi Sankyo and the investigator reserve the right to terminate the study according to the study contract. The investigator should notify the IEC/IRB in writing of the study's completion or early termination and send a copy of the notification to Daiichi Sankyo.

Subjects may be eligible for continued treatment with investigational product by extension protocol or as provided for by the local country's regulatory mechanism. However, Daiichi Sankyo reserves the unilateral right to determine whether to supply the investigational product, and by what mechanism, after termination of the trial and before it is available commercially.

7.5. Study Documentation and Archival

The investigator should maintain a list of appropriately qualified persons to whom he/she has delegated study duties. All persons authorized to make entries and/or corrections on case report forms will be included on the Delegation of Authority Form.

Source documents are original documents, data, and records from which the subject's case report form data are obtained. These include but are not limited to hospital records, clinical and office charts, laboratory and pharmacy records, diaries, microfiches, radiographs, and correspondence. Case report form entries may be considered source data if the CRF is the site of the original recording (ie, there is no other written or electronic record of data). In this study, case report forms calculating IPSS may be used as source documents for IPSS risk category assignment.

The investigator and study staff are responsible for maintaining a comprehensive and centralized filing system of all study-related (essential) documentation, suitable for inspection at any time by representatives from Daiichi Sankyo and/or applicable regulatory authorities. Elements should include:

- Subject files containing completed case report forms, informed consent forms, and subject identification list

- Study files containing the protocol with all amendments, investigator's brochure, copies of prestudy documentation and all correspondence to and from the IEC/IRB and Daiichi Sankyo.

- Proof of receipt, Investigational Product Accountability Record, Return of Investigational Product for Destruction, Final Investigational Product Reconciliation Statement, and all drug-related correspondence. *. All expired/unused study drug will be destroyed per the institution policy.In addition, all original source documents supporting entries in the case report forms must be maintained and be readily available. No study document should be destroyed without prior written agreement between Daiichi Sankyo and the investigator. Should the investigator wish to assign the study records to another party or move them to another location, he/she must notify Daiichi Sankyo in writing of the new responsible person and/or the new location.

7.6. Investigator Communication with Supporting Company:

The Alliance Pharmacovigilance Agreement (Section VIII. Safety Information Management) executed in February 2018 describes all Safety Information-related exchange obligations and will govern all ICSR case report exchanges. ICSR case reports as defined in this section will be exchanged as per the timelines stated and sent to CSPV-Clincial@dsi.com via secure e-mail. Additional information secured after the initial exchange will be sent as follow-up report. A final report to document resolution of the SAE is also required.

7.7. Product Complaints

In addition to compliance with all FDA requirements pursuant to 21 CFR 211 and 21 CFR 820, Principal Investigator will report to Daiichi Sankyo within 24 hours any suspected quality defect in an Daiichi Sankyo Product or its Daiichi Sankyo-provided packaging, labeling, or medical device component (collectively, "Product Complaint". Principal Investigator will report Product Complaints that involve a Daiichi Sankyo Product, whether Daiichi Sankyo has supplied the Daiichi Sankyo Product used in the Study or not. Daiichi Sankyo's contact for reporting Product Complaints shall be handled according to the Quality Agreement included in the study binder by contacting Daichi Sankyo, INC., office 211 Mt. Airy Road, Basking Ridge, NJ 07920-2311. (908) 992-7072 or email: mreeves@dsi.com.

8. STATISTICAL CONSIDERATIONS

8.1 Statistical Design

This is a phase 1/2 study of DS-3032b with LDAC, with or without addition of venetoclax in patients with AML. The primary objective of the study in phase I is to evaluate the safety and tolerability of the combination therapy and determine the maximum tolerated dose (MTD). The primary objective of the phase II part is to evaluate the efficacy of this combination treatment in newly diagnosed and relapsed/refractory AML patients, respectively. An estimated 58 patients will be enrolled.

8.1.1: Phase I

In this phase, the 3+3 algorithm will be applied for dose escalation or dose de-escalation. There are 3 doses involved (Table 8.1). The starting dose tested will be dose level 0, and escalate to planned dose

level +2 if dose level 0, +1, are determined safe. Initially three patients will be treated at dose level 0. Up to 18 patients will be enrolled in the phase I part.

Table 8.1 Phase 1 dose reduction and escalation for DS-3032b + LDAC + venetoclax

Dose Level	Cytarabine (mg BID), SubQ	Venetoclax (mg/daily), oral	DS-3032b (mg/daily), oral
+2	20mg BID x 7 days (D1-10)	600 mg daily x 14 days (D1-14)	260mg daily x D5-7 and D15-17
+1	2020mg BID x 7 days (D1-10)	600mg daily x 14 days (D1-14)	200mg daily x 7 days (D8-14)
0 (starting dose)	20mg BID x 7 days (D1-7)	----	120mg daily x 14 days (D8-21)
-1	20mg BID x 7 days (D1-7)	----	120mg daily x 7 days (D8-14)

The following is a description of dose escalation rule.

- If none of the initial 3 patients in the cohort experience a DLT, then a new cohort of 3 patients will be treated at the next higher dose level (i.e. Dose Level 1).
- If 1 of the 3 initial patients experiences a DLT, then treat 3 additional patients at the same dose (i.e. Dose Level 0). Escalation will only continue if 1 of the 6 patients experiences a DLT.
- If ≥2/3 or ≥2/6 patients experience DLTs in the first treatment cycle, the MTD has been exceeded. No additional patients will be treated at that dose and no further dose escalation above that dose either will occur. A new cohort of three patients will be enrolled at the next lower dose if at that dose only three patients were treated.
- If dose level -1 is too toxic (i.e. ≥2/3 or ≥2/6 patients experience DLTs in the first cycle), the study will be stopped. MTD is defined the highest dose at which no more than one patient out of 6 patients experience DLTs in the first cycle. Table 8.2 lists the probabilities of dose escalation based on different DLT rate for the 3+3 design.

- **Table 8.2** below gives the probabilities of dose escalation based on true DLT risk in the 3+3 design.

	True DLT rate								
	10%	20%	30%	40%	50%	60%	70%	80%	90%
Probability of escalation	0.91	0.71	0.49	0.31	0.17	0.08	0.03	0.01	0.001

-
- **8.1.2. Phase II:**
- The Phase II portion is designed to determine the response rate of the combination of DS-3032b and LDAC, with or without the addition of venetoclax (if the dose level 0 is identified as MTD,

then the phase II patients will be treated only with combined DS-3032b and LDAC without venetoclax) in two populations of 20 patients each:

- (Arm A): Newly diagnosed patients with AML;
- (Arm B): Relapsed/refractory AML patients.
-
- The primary endpoint for the phase II part is overall response rate (ORR) which will be evaluated within the first 2 cycles of the treatment.
-
- **Arm A:** The historical data suggests an ORR rate of 25% for this population and the target ORR with DS-3032b and LDAC will be $\geq$ 40%. This treatment combination will be considered worthy of further investigation if it elicits an increase in ORR to 40%, with acceptable toxicity.

- Given this, we will stop enrollment if the observed patients' data suggest that:

- Pr ($ORR_E > ORR_H + 0.15 | data$) < 0.02 or
- Pr ($TOX_E > 0.30 | data$) > 0.88

Here ORR_E and ORR_H are the ORR rate for new experimental therapy and the historical treatment, respectively. TOX_E is the toxicity rate which is defined as therapy related non-hematological grade 3/4 toxicity within the 1st cycle of treatment. That is, if at any time during the study we determine that there is a less than 2% chance that the average ORR rate improves over historical ORR rate by more than 15% we will stop enrollment to this cohort. The second condition will stop the study early if excessive therapy-related non-hematological grade 3/4 toxicity (>30%) is highly probable (i.e., probability >88%). The ORR_E and ORR_H are assumed to follow a prior of Beta (0.5, 1.5) and a constant of 25%, respectively. The stopping boundaries for ORR, based on these assumptions and monitoring conditions are found in **Table 8.3.** We will apply these stopping boundaries continuously starting from the fifth patient in a cohort size of 5 patients. For example, accrual will cease if 1 patient experiences ORR among the first 10 patients treated.

Table 8.3. Stopping boundaries for ORR rate for the cohort with Newly diagnosed patients with AML

# of patients	Stop the trial if there are this many ORR totals
5	0
10	0-1
15	0-2
20	Always stop with this many patients

The monitoring rule for the toxicity rate, based on these assumptions and monitoring conditions above is found in **Table 8.4**. For example, accrual will cease if 4 or more patients experience toxicities among the first 5 patients.

Table 8.4. Stopping boundaries for Toxicity for the cohort with Newly diagnosed patients with AML

# of patients	Stop the cohort if there are this many therapy-related non-hematological grade 3/4 toxicities total
5	4-5
10	6-10
15	7-15
20	Always stop with this many patients

Multc Lean Desktop (version 2.1.0) was used to generate the toxicity and futility stopping boundaries and the OC table (Table 8.5). A 30% toxicity constant rate and beta (0.6, 1.4) priors were assumed for the standard treatment toxicity constant rate and experimental treatment toxicity prior distribution, respectively.

The probability of stopping the study early if the true ORR of the combination treatment was 40% and the true toxicity rate was 30% was 24%. Probabilities of stopping early for high true toxicity rates (i.e., 50%) were 74% when the true ORR was 40% and 72% when true ORR rate was 50%.

Table 8.5. Operating characteristics for simultaneous monitoring response and toxicity rates for patients treated with combination treatment for the cohort with Newly diagnosed patients with AML

True Toxicity Rate	True ORR	Prob(stop the trial early)
0.20	0.15	0.72
	0.25	0.40
	0.4	0.13
	0.5	0.06
0.30	0.15	0.75
	0.25	0.47
	0.4	**0.24**
	0.5	0.18
0.50	0.15	0.92
	0.25	0.82

Table 8.5. Operating characteristics for simultaneous monitoring response and toxicity rates for patients treated with combination treatment for the cohort with Newly diagnosed patients with AML		
True Toxicity Rate	True ORR	Prob(stop the trial early)
	0.4	0.74
	0.5	0.72

If the study is not stopped early and 20 patients have been treated and evaluated in the study, assuming 8 of the 20 patients achieve ORR under the combination treatment and without using any non-informative prior distribution, then the 95% credible interval for ORR rate will be (0.20 -0.62).

Arm B: The historical data suggests an ORR rate of 15% for this population and the target ORR with DS-3032b and LDAC will be $\geq$ 30%. This treatment combination will be considered worthy of further investigation if it elicits an increase in ORR to 30%, with acceptable toxicity.

Given this, we will stop enrollment if the observed patients' data suggest that:

1) Pr ($ORR_E > ORR_H + 0.15 | data$) < 0.02 or
2) Pr ($TOX_E > 0.30 | data$) > 0.88

Here ORR_E and ORR_H are the ORR rate for new experimental therapy and the historical treatment, respectively. TOX_E is the toxicity rate which is defined as therapy related non-hematological grade 3 or higher toxicity within the 1st cycle of treatment. That is, if at any time during the study we determine that there is a less than 2% chance that the average ORR rate improves over historical ORR rate by more than 15% we will stop enrollment to this cohort. The second condition will stop the study early if excessive therapy-related non-hematological grade 3/4 toxicity (>30%) is highly probable (i.e., probability >88%). The ORR_E and ORR_H are assumed to follow a prior of Beta (0.3, 1.7) and a constant of 15%, respectively. The stopping boundaries for ORR, based on these assumptions and monitoring conditions are found in **Table 8.6.** We will apply these stopping boundaries continuously starting from the fifth patient in a cohort size of 5 patients. For example, accrual will cease if 0 patient experiences ORR among the first 10 patients treated.

Table 8.6. Stopping boundaries for ORR rate

# of patients	Stop the trial if there are this many ORR totals
5-10	0
15	0-1

20	Always stop with this many patients

The monitoring rule for the toxicity rate, based on these assumptions and monitoring conditions above is found in **Table 8.7**. For example, accrual will cease if 4 or more patients experience toxicities among the first 5 patients.

Table 8.7. Stopping boundaries for Toxicity

# of patients	Stop the cohort if there are this many therapy-related non-hematological grade 3/4 toxicities total
5	4-5
10	6-10
15	7-15
20	Always stop with this many patients

Multc Lean Desktop (version 2.1.0) was used to generate the toxicity and futility stopping boundaries and the OC table (Table 8.8). A 30% toxicity constant rate and beta (0.6, 1.4) priors were assumed for the standard treatment toxicity constant rate and experimental treatment toxicity prior distribution, respectively.

The probability of stopping the study early if the true ORR of the combination treatment was 30% and the true toxicity rate was 30% was 30%. Probabilities of stopping early for high true toxicity rates (i.e., 50%) were 76% when the true ORR was 30% and 72% when true ORR rate was 50%.

Table 8.8. Operating characteristics for simultaneous monitoring response and toxicity rates

True Toxicity Rate	True ORR	Prob(stop the trial early)
0.20	0.15	0.53
	0.25	0.28
	0.3	0.20
	0.5	0.05
0.30	0.15	0.59
	0.25	0.37
	0.3	0.30
	0.5	0.17

Table 8.8. Operating characteristics for simultaneous monitoring response and toxicity rates		
True Toxicity Rate	True ORR	Prob(stop the trial early)
0.50	0.15	0.86
	0.25	0.79
	0.3	0.76
	0.5	0.72

If the study is not stopped early and 20 patients have been treated and evaluated in the study, assuming 6 of the 20 patients achieve ORR under the combination treatment and without using any non-informative prior distribution, then the 95% credible interval for ORR rate will be (0.13 -0.51).

8.2 Sample Size

Up to 58 patients may be entered in this trial. The phase I portion is anticipated to enroll 18 patients, unless the lowest dose is too toxic. The phase II portion will evaluate 40 patients.

8.3 Analysis Populations:

8.3.1 Safety Population:

All patients who receive at least 1 dose of study treatment will be included in the analysis of safety regardless of the duration of treatment. Patients who experience adverse events during the Screening period but who do not start on study treatment due to reasons that include, but are not limited to ineligibility/screen failure, death or withdrawal of consent, will not be included in the safety population.

8.3.2 DLT-evaluable Population (Phase I portion only):

Unless doses are missed in Cycle 1 due to DLT(s), a patient must receive at least 85% of the planned doses of DS-3032b to be considered evaluable for DLTs. If a patient received fewer doses/days of treatment in the first cycle of treatment for reasons other than a DLT, the patient will be considered non-evaluable for DLT and replaced.

8.4 Statistical Analysis Plan:

The rates of response to therapy (ORR) will be estimated along with the 95% credible interval. Fisher's exact test and Wilcoxon rank test will be used in the data analyses of categorical and continuous variables, respectively. The Kaplan-Meier method will be used to estimate the probabilities of event free survival (EFS), DOR, and overall survival (OS) with appropriate levels of error as indicated, such as 95% confidence intervals for the medians or standard errors for the proportions surviving at a specific time. The duration of response is defined as the number of days from the date of initial response to the date of first documented disease progression/relapse or death, whichever occurs first. Event-free survival is defined as the number of days from the date of treatment initiation to the date of documented treatment failure, relapses from CR, or death from any cause, whichever occurs first, and will be calculated for all patients. The overall survival is defined as the time from treatment start till death or last follow-up. Log-rank tests will be used to compare among subgroups of patients in terms of EFS or OS if any subgroups are large enough with enough events to warrant a comparison. Anti-tumor activity, PD markers, exploratory biomarkers and drug exposure levels will be summarized graphically and with descriptive statistics. Exact logistic regression analysis will be used to evaluate the association of PD/PK parameters with response. Safety data will be summarized using frequency and percentage, by category and severity. Exploratory measures of MRD and genetic expression will be presented graphically with descriptive statistics to identify potential prognostic markers for inclusion in future trials.

The Investigator is responsible for completing toxicity/efficacy summary reports and submitting them to the IND office Medical Affairs and Safety Group for review and approval. These should be submitted as follows:

- Phase I:

After the first 3 evaluable patients complete cycle 1 of study treatment, and every 3 evaluable patients thereafter, prior to advancing/changing dose levels.

- Phase II:

After the first 5 evaluable patients complete cycle 1 of study treatment, and every 5 patients thereafter, until enrollment is complete.

A copy of the cohort summary should be placed in the Investigator's Regulatory Binder under "sponsor correspondence".

9.0 Protocol Language Template Outside Physician Participation During Treatment

1. MDACC Physician communication with the outside physician is required prior to the patient returning to the local physician. This will be documented in the patient record.

2. A letter to the local physician outlining the patient's participation in a clinical trial will request local physician agreement to supervise the patient's care.

3. Protocol required evaluations outside MDACC will be documented by telephone, fax or e-mail. Fax and/or e-mail will be dated and signed by the MDACC physician, indicating that they have reviewed it.

4. Changes in drug dose and/or schedule must be discussed with and approved by the MDACC physician investigator, or their representative prior to initiation, and will be documented in the patient record.

5. A copy of the informed consent, protocol abstract, treatment schema and evaluation during treatment will be provided to the local physician.

6. Documentation to be provided by the local physician will include drug administration records, progress notes, reports of protocol required laboratory and diagnostic studies and documentation of any hospitalizations.

7. The home physician will be requested to report to the MDACC physician investigator all life threatening events within 24 hours of documented occurrence.

8. Patients will return to MDACC every month for evaluation.

Appendix A: Recommendations for initial management of electrolyte imbalances and prevention of tumor lysis syndrome (TLS)_

**Abnormality**	**Management Recommendations**
**Hyperkalemia (including rapidly rising potassium)**	
Potassium > upper limit of normal	• Perform STAT ECG and commence telemetry.<br>• Nephrology (or other acute dialysis service) notification with consideration of initiating dialysis.<br>• Administer Kayexalate 60 g (or Resonium A 60 g).<br>• Administer furosemide 20 mg IV × 1.<br>• Administer calcium gluconate 100 – 200 mg/kg IV slowly if there is ECG/telemetry evidence of life-threatening arrhythmias.<br>• Recheck potassium, phosphorus, uric acid, calcium and creatinine in 1 hour STAT.
**Hyperuricemia**	
Uric acid ≥ 10 mg/dL (595 µmol/L)<br>OR<br>Uric acid ≥ 8.0 mg/dL (476 µmol/L) with 25% increase and creatinine increase ≥ 0.3 mg/dL (≥ 0.027 mmol/L) from pre-dose level	• Administer rasburicase (dose per institutional guidelines).<br>• If rasburicase is used, sodium bicarbonate should not be used as this may exacerbate calcium phosphate precipitation.<br>• Notify nephrology (or other acute dialysis service).<br>• Recheck potassium, phosphorus, uric acid, calcium and creatinine in 1 hour STAT.<br>• If uric acid < 8.0 mg/dL 1 hour later, repeat potassium, phosphorus, uric acid, calcium and creatinine 2 and 4 hours later, if no other evidence of tumor lysis.
**Hypocalcemia**	
Calcium ≤ 7.0 mg/dL (1.75 mmol/L)<br>AND<br>Patient symptomatic e.g., muscle cramps, hypotension, tetany, cardiac arrhythmias)	• Administer calcium gluconate 50 – 100 mg/kg IV slowly with ECG monitoring.<br>• Recheck potassium, phosphorus, uric acid, calcium and creatinine in 1 hour STAT.<br>• If calcium normalized 1 hour later, repeat potassium, phosphorus, uric acid, calcium and creatinine 2 and 4 hours later, if no other evidence of tumor lysis.<br>• Calculate corrected calcium and check ionized calcium if albumin low.
**Hyperphosphatemia**	
Phosphorus ≥ 6.0 mg/dL with ≥ 0.5 mg/dL increase	• Administer a phosphate binder (e.g., aluminum hydroxide, calcium carbonate, sevelamer hydroxide, or lanthanum carbonate).<br>• Nephrology (or other acute dialysis service) notification (dialysis required for phosphorus ≥ 10 mg/dL).<br>• Recheck potassium, phosphorus, uric acid, calcium and creatinine in 1 hour STAT.
**Creatinine**	
Increase ≥ 25% from baseline	Start or increase rate of IV fluids.<br>Recheck potassium, phosphorus, uric acid, calcium and creatinine in 1 – 2 hours STAT.

References:

1. Estey, E.H., *How I treat older patients with AML.* Blood, 2000. **96**(5): p. 1670-3.
2. Goldstone, A.H., et al., *Attempts to improve treatment outcomes in acute myeloid leukemia (AML) in older patients: the results of the United Kingdom Medical Research Council AML11 trial.* Blood, 2001. **98**(5): p. 1302-11.
3. Grimwade, D., et al., *The predictive value of hierarchical cytogenetic classification in older adults with acute myeloid leukemia (AML): analysis of 1065 patients entered into the United Kingdom Medical Research Council AML11 trial.* Blood, 2001. **98**(5): p. 1312-20.
4. Momand, J., et al., *The mdm-2 oncogene product forms a complex with the p53 protein and inhibits p53-mediated transactivation.* Cell, 1992. **69**(7): p. 1237-45.
5. Vassilev, L.T., et al., *In vivo activation of the p53 pathway by small-molecule antagonists of MDM2.* Science, 2004. **303**(5659): p. 844-8.
6. Shangary, S., et al., *Temporal activation of p53 by a specific MDM2 inhibitor is selectively toxic to tumors and leads to complete tumor growth inhibition.* Proc Natl Acad Sci U S A, 2008. **105**(10): p. 3933-8.
7. Shangary, S. and S. Wang, *Small-molecule inhibitors of the MDM2-p53 protein-protein interaction to reactivate p53 function: a novel approach for cancer therapy.* Annu Rev Pharmacol Toxicol, 2009. **49**: p. 223-41.
8. Vassilev, L.T., *MDM2 inhibitors for cancer therapy.* Trends Mol Med, 2007. **13**(1): p. 23-31.
9. Mrinal M. Gounder, T.M.B., Gary K. Schwartz, et al, *A phase 1 study of MDM2 inhibitor DS-3032b in patients with advanced solid tumors and lymphomas.* ASCO abstract 2581, 2016.
10. Courtney D. DiNardo, J.R., Michael Andreeff, et al., *Phase 1 dose escalation study of MDM2 inhibitor DS-3032b in patients with hematological malignancies - preliminary results.* Blood, 2016. **128:593**.
11. Souers, A.J., et al., *ABT-199, a potent and selective BCL-2 inhibitor, achieves antitumor activity while sparing platelets.* Nat Med, 2013. **19**(2): p. 202-8.
12. Konopleva, M., et al., *Efficacy and Biological Correlates of Response in a Phase II Study of Venetoclax Monotherapy in Patients with Acute Myelogenous Leukemia.* Cancer Discov, 2016. **6**(10): p. 1106-1117.
13. Bogenberger, J.M., et al., *Ex vivo activity of BCL-2 family inhibitors ABT-199 and ABT-737 combined with 5-azacytidine in myeloid malignancies.* Leuk Lymphoma, 2015. **56**(1): p. 226-9.
14. Konopleva, M., et al., *The anti-apoptotic genes Bcl-X(L) and Bcl-2 are over-expressed and contribute to chemoresistance of non-proliferating leukaemic CD34+ cells.* Br J Haematol, 2002. **118**(2): p. 521-34.
15. Pan, R., et al., *Selective BCL-2 inhibition by ABT-199 causes on-target cell death in acute myeloid leukemia.* Cancer Discov, 2014. **4**(3): p. 362-75.
16. Tsao, T., et al., *Concomitant inhibition of DNA methyltransferase and BCL-2 protein function synergistically induce mitochondrial apoptosis in acute myelogenous leukemia cells.* Ann Hematol, 2012. **91**(12): p. 1861-70.
17. Andrew Wei, S.A.S., Gail J. Roboz, Jing-Zhou Hou, Walter Fiedler, Tara L. Lin, Roland B. Walter, Anoop Enjeti, Brenda Chyla, Relja Popovic, Kaffa Fakouhi, Pooja Shah, Martin Dunbar, Tu Xu, Mack Mabry and John Hayslip, *Phase 1/2 Study of Venetoclax with Low-Dose Cytarabine in Treatment-Naive, Elderly Patients with Acute Myeloid Leukemia Unfit for Intensive Chemotherapy: 1-Year Outcomes.* Blood 130:890;, 2017.
18. DiNardo, C.D., et al., *Venetoclax combined with decitabine or azacitidine in treatment-naive, elderly patients with acute myeloid leukemia.* Blood, 2019. **133**(1): p. 7-17.

19. DiNardo, C.D., et al., *Clinical experience with the BCL2-inhibitor venetoclax in combination therapy for relapsed and refractory acute myeloid leukemia and related myeloid malignancies.* Am J Hematol, 2018. **93**(3): p. 401-407.
